# Supplementary material for: Low pressure amide hydrogenation enabled by magnetocatalysis
Source: Nat Commun. 2025 Apr 11;16:3464. doi: 10.1038/s41467-025-58713-6 (PMC11992221; doi:10.1038/s41467-025-58713-6)
Supplement: Supplementary file 1 — Supplementary Information [file 41467_2025_58713_MOESM1_ESM.pdf]

Supporting Information

LOW PRESSURE AMIDE HYDROGENATION ENABLED BY MAGNETOCATALYSIS

Sheng-Hsiang Lin[1,2,‡], Sihana Ahmedi[1,2,‡], Carlotta Campalani[1], Aaron Kretschmer[1], Yves Kayser[1], Liqun Kang[1], Serena DeBeer[1], Walter Leitner[1,2], Alexis Bordet[1]*

[1] Max Planck Institute for Chemical Energy Conversion, Stiftstraße 34-36, 45470, Mülheim an der Ruhr, Germany

[2] Institute of Technical and Macromolecular Chemistry, RWTH Aachen University, 52074 Aachen, Germany

‡ Equal contribution

*Corresponding author: alexis.bordet@cec.mpg.de

Table of Content

Materials and Methods **2**

Materials 2

Characterization Techniques 2

Product Analysis 4

Synthesis and Characterization 5

Catalytic Experiments 6

Energy consumption analysis 7

Supplemental Tables **9**

Supplementary Figures **12**

Isolated Yields **17**

GC-FID Chromatograms of crude mixtures **20**

References **41**

Materials and Methods

Materials

All syntheses were performed under argon either by using Schlenk techniques or in a glove box. Solvents were purified through a solvent purification system (MBraun-SPS-7) or dried over activated 4 Å molecular sieves then degassed and preserved under an argon atmosphere before use. Hexadecylamine (HDA, 99%), palmitic acid (PA, 99%) and platinum on alumina material (Pt/Al_2O_3, 1 wt% Pt) were purchased from Sigma-Aldrich. Amides are purchased from the local suppliers (e.g. Sigma-Aldrich, abcr, Alfa Aesar) and used without further purification. The bis[bis(trimethylsilyl)amido]iron(II) ($\{Fe[N(SiMe_3)_2]_2\}_2$) was synthesized following the literature.[1]

Characterization Techniques

- SEM and EDX measurements were carried out using a Hitachi S-5500.
- XRD measurements were performed on a PANalytical Empyrean diffractometer using Co $K\alpha$ radiation (λ = 0.1789 nm) at 45 kV and 40 mA.
- ^{57}Fe Mössbauer spectra were collected on a spectrometer with conventional constant acceleration of the γ source (^{57}Co source in Rh matrix, 1.8 GBq). The sample temperature was kept constant using a Cryogen-Free Magnet (CFM) with integrated variable temperature insert (VTI) for zero-field measurements. The minimum experimental linewidth was 0.24 $mm \cdot s^{-1}$. Isomer shifts are quoted relative to α-iron at 300 K. The ^{57}Fe Mössbauer spectra were simulated and fitted with *MX* program written by Dr. Eckhard Bill.
- Superconducting quantum interference device (SQUID) data were collected on a Quantum Design MPMS-3 SQUID magnetometer. DC susceptibility was recorded at 300 K with an applied DC field of 1 T, if not stated otherwise. The SQUID data analysis was conducted with *JulX2* program written by Dr. Eckhard Bill.
- Fe K-edge data was collected using an easyXES-100 spectrometer in transmission mode.[2] An X-ray tube with a W anode set to 25 kV and 2 mA, a 1 mm wide entrance slit, a Ge (310) crystal in second diffraction order and a silicon drift detector were used to this end. The samples were enclosed in an in-house designed anaerobic sample cell sealed with Kapton films that served as entrance and exit windows for

the X-rays. The X-ray transmission of the Kapton films was included in the reference measurement without sample. The offset on the energy scale of the spectrometer was determined using an Fe foil with a thickness of 4 µm. The energy scan was repeated 60 times with an integration time of 1s per position in each scan. The Pt L_3-edge XAFS spectra for the fresh and spent catalysts were collected at the P65 beamline of PETRA III (P65 applied X-ray absorption spectroscopy) in fluorescence mode due to the low relative Pt concentration.[3] This aspect and the low flux of the source made it unconceivable to extract meaningful data in a reasonable time scale with the in-house based X-ray spectrometer. At the P65 beamline, synchrotron radiation from the 3rd harmonic radiation of an 11-period undulator and monochromatized by a Si(111) double crystal monochromator (DCM) was used. The DCM was operated in QEXAFS mode, and the undulator energy offset to the DCM was calibrated to have the maximum photon flux. Rh coated mirrors were used for focusing and collimation. The beam size at the sample position was approx. 0.5 x 1.0 mm^2 (V x H) and the photon flux was ~10^{11} photons/s (without attenuation). The incident beam intensity was monitored by an ionization chamber (4 cm length, filled with 680 mbar N_2 and 370 mbar $Ar_{(g)}$) and the fluorescence signal was detected by a 4-element silicon drift detector (SDD). A 3 µm thick V foil was mounted in front of the fluorescence detector in order to avoid excessively high dead time due to significant Fe $K\alpha$ and $K\beta$ fluorescence from the sample. The sample pellets were prepared inside a glovebox and mounted into in-house designed fluorescence sample cells to prevent exposure to air or moisture. The measurements were performed at room temperature. The XAFS of each sample was measured 5 times and merged to improve the signal-noise ratio. A Pt foil was measured separately as the reference for energy calibration. The energy of the incident beam was calibrated by assigning the energy of the first inflection in the first derivative XANES of Pt foil to 11564 eV.

- The Pt L_3-edge XAFS spectra were analyzed using the Demeter software package (including Athena and Artemis programs, version 0.9.26).[4] Pre-edge background subtraction and post-edge normalization of the XAFS data were performed using the Athena program. A linear regression background in the range of 11481 eV to

11497 eV was determined, and a quadratic polynomial regression for post-edge normalization in the range of 11598 to 12306 eV was applied. The fitting of EXAFS spectra (R range: 1.2 to 3.2 Å, k-range: 3.0 to 12.3 $Å^{-1}$) was performed using the Artemis program based on scattering paths generated from FEFF6. The amplitude reduction factor S_0^2 is determined to be 0.785 by fitting of k^2-weighted R-space EXAFS of the Pt foil based on the standard crystal parameters of platinum metal (retrieved from Crystal Open Database, entry ID: 9008480), and was used as fixed parameter in the EXAFS fitting model for the catalysts.

- High resolution aberration-corrected BF-STEM and HAADF-STEM images were acquired using a probe-corrected (CEOS) JEOL ARM300CF electron microscope (instrument E02) in the electron Physical Science Imaging Center (ePSIC) at Diamond Light Source (DLS, UK). The acceleration voltage was 80 kV and the probe size was set to 8C (spot 8) with a 30 µm probe-forming aperture (CL aperture) selected, resulting in a probe convergence semi-angle of 24.8 mrad and a beam current of 28.3 pA. The STEM camera length was set to 9.0 cm, which allowed the ADF detector to integrate the scattered electron intensity between 73.7 ± 1.8 and 155.4 ± 1.8 mrad. In addition, a 3 mm aperture was inserted for the BF imaging, corresponding to a semi-angle of 14.8 ± 1.2 mrad (outer angle) for the BF detector. For each sample, a small amount of dry powder was sprinkled on a 200-mesh Cu grid with lacey carbon support film. Each sample was exposed to an intense electron beam for 10-15 minutes ('beam shower') to eliminate the accumulation of carbon contamination during the STEM imaging. Gatan Microscopy Suite software was used for image data acquisition.

Product Analysis

Product analysis was done by GC-FID (gas chromatography coupled with flame ionization detection) on a Shimadzu GC 2030 equipped with a CP-WAX-52CB column and further by GC-MS (gas chromatography coupled with mass spectrometry) on a Shimadzu QP 2020 instrument. Product quantification was done by referencing the product peak area to the peak area of the added tetradecane standard, following internal GC calibration with the isolated products. For identification of unknown products and trace compounds GC-

MS was used with its internal compound library for product identification. NMR spectra were recorded on Bruker AV-400 spectrometer. The coupling constants (*J*) are given in Hertz (Hz), and the chemical shifts (δ) expressed in ppm are calibrated using deuterated solvent ($CDCl_3$ at 7.26 ppm for ^1H NMR, and 77.2 ppm for ^{13}C NMR). The peak patterns are indicated as follows: s = singlet; d = doublet; t = triplet; m = multiplet.

Synthesis and Characterization

Synthesis of ICNPs

Iron carbide nanoparticles (ICNPs) were prepared following a previously reported procedure.[5] They were obtained in two steps through the carbidization of preformed Fe(0) nanoparticles:

Fe(0) NPs

In the glove box, 0.65 mmol of PA (333.2 mg) and 0.5 mmol of HDA (241.5 mg) were independently dissolved in 10 mL mesitylene and added sequentially to a green solution of 0.5 mmol $\{Fe[N(SiMe_3)_2]_2\}_2$ (376.5 mg) in 20 mL mesitylene in a Fisher-Porter (FP) bottle. The FP bottle was then pressurized with H_2 (2 bar) and placed in an oil bath at 150°C for 48 h under vigorous magnetic stirring. After 48 h, the reaction was stopped and the NPs were recovered by decantation assisted by a magnet, and washed 3 times (3x10 mL) with toluene and 3 times (3x10 mL) with THF. The NPs were then dried under vacuum.

Carbidization of Fe(0) NPs

In the glovebox, Fe(0) NPs (50 mg, 0.45 mmol of iron) were dispersed in mesitylene (9 mL) in a FP bottle, and the mixture was pressurized with CO/H_2 (2 bar / 2 bar) at 150°C for 120 h. At the end of the reaction, the NPs were recovered by decantation assisted by a magnet and were washed 3 times with toluene (3x5 mL). The NPs (ca. 75 wt% Fe) were then dried under vacuum.

Synthesis of ICNPs@Pt/Al_2O_3 (28.5 wt% ICNPs loading)

In a typical experiment, ICNPs (15.0 nm; 10.0 mg) and Pt/Al_2O_3 (25.0 mg, 1 wt% Pt) were dispersed in THF (1.0 mL) in a FP bottle. The bottle was then sealed under an argon atmosphere and subjected to sonication for 1 minutes. At the end of the impregnation step, a black precipitate and a clear supernatant were observed. To finish, the magnetic powder was dried under vacuum and treated using magnetic induction (μ_0H_{max} = 45 mT, 350 kHz) for 1 hour to anchor the ICNPs to the Al_2O_3 surface and prevent leaching.

Catalytic Experiments

Magnetocatalytic experiments (i.e. with magnetic induction heating)

In a typical experiment, ICNPs@Pt/Al_2O_3 (35.0 mg, 1.26 μmol Pt), solvent (0.5 mL), and the substrate (0.10 mmol) were placed in a FP bottle. The FP bottle was flushed, and pressurized with the desired pressure of hydrogen (3 bar). The reaction mixture was placed at the center of a copper coil at the desired magnetic field amplitude and fixed frequency of 350 kHz. Once the reaction was finished, the reactor was cooled and vented. After filtration, the reaction mixture was analyzed by GC-FID using tetradecane as the internal standard.

Catalysis with conventional heating

In a typical experiment, the catalyst, solvent (0.5 mL), and substrate (0.10 mmol) were placed in a FP bottle. The FP bottle was flushed, and pressurized with the desired pressure of hydrogen (3 bar). The reaction mixture placed in an oil bath and the reaction performed at the desired temperature. Once the reaction was finished, the FP bottle was cooled and vented. After filtration, the reaction mixture was analyzed by GC-FID using tetradecane as the internal standard.

For reactions performed at higher H_2 pressure (50 bar H_2), stainless steel autoclaves heated in aluminum heating blocks were used.

Kinetic study

Time profiles were collected following similar protocols, with individual reactions for each selected time.

Recycling experiments

In a typical experiment, ICNPs@Pt/Al_2O_3 (35.0 mg, 1.26 μmol Pt), decalin (0.5 mL), and 1-acetyl-3-methylpiperidine (12.9 mg, 0.10 mmol) were placed in a Fisher-Porter bottle. The Fisher-Porter bottle was flushed, and pressurized with the desired pressure of hydrogen (3 bar). The reaction mixture was placed at the center of a copper coil at 72 mT and 350 kHz for 0.5 hour. Once the reaction was finished, the reactor was cooled and vented. After filtration, the reaction mixture was analyzed by GC-FID using tetradecane as the internal standard. For the next cycle, fresh portions of the substrate (0.10 mmol) and decalin (0.5 mL) were added and the reaction mixture was performed again. This procedure was repeated for each catalyst cycle by pressurizing the Fisher-Porter bottle with 3 bar of hydrogen.

Energy consumption analysis

With magnetocatalysis (72 mT), the ICNPs on ICNPs@Pt/Al_2O_3 absorb ca. 98 W[a] (or 98 J s^{-1}) of power input and release it as thermal energy, accounting for ca. 1.4 MJ of energy over 4 hours. Importantly, the catalyst reached almost instantaneously its working temperature. Under these conditions (72 mT, 350 kHz, 3 bar H_2, 4 h), substrate **1** was fully converted, and product **1a** was obtained in quantitative yield. Strikingly, with conventional heating, the oil bath took 100 min to reach the target 200 °C, consuming ca. 3.6 MJ of energy as measured by a power meter. Operating it for 4 hours at 200 °C consumed another 8.6 MJ of energy, while catalytic performance remained poor (7% conversion, 7% **1a**). Thus, for much lower catalytic performance, conventional heating consumed ca. 12.2 MJ, while the magnetically heated catalyst consumed only 1.4 MJ (see Table below for a summary).

	**Magnetic (72 mT, 350 kHz)**	**Conventional (200 °C)**
Time to target T°C (h)	**0**	**1.67**
Reactor T°C	**156**	**200**
Reaction time (h)	4	4
Energy input to reactor (MJ)	**1.4**	**12.2**
Yield of **1a** (%)	**>99**	**7**
Energy efficiency toward product formation (mmol MJ^{-1})	**0.07**	**0.0006**

[a]Heat generation by ICNPs under ACMF:

35 mg ICNPs@Pt/Al_2O_3 catalyst => 7 mg Fe

SAR_{ICNPs} = ca. 4000 W/g_{Fe} at 72 mT 100 kHz => ca. 14000 W/g_{Fe} at 72 mT and 350 kHz (approximation following the Stoner-Wohlfarth model)

=> **98 W** of heat released by the ICNPs in the reactor => **98 J s^{-1}**, equivalent to **1.4 MJ** in 4 h**.**

[ref] H. Kreissl, J. Jin, S.-H. Lin, D. Schüette, S. Störtte, N. Levin, B. Chaudret, A. J. Vorholt, A. Bordet, W. Leitner, *Angew. Chem. Int. Ed.* **2021**, *60*, 26639.

Supplemental Tables

Table S1. Summary of state-of-the-art heterogenous catalysts used for the hydrogenation of *N*-acetylpiperidine to *N*-ethylpiperidine. Temp. = temperature. P. = pressure. Conv. = conversion. Select. = selectivity. Ref. = reference. HBEA and HMFI = zeolites. HAP = hydroxyapatite. N/A = not applicable.

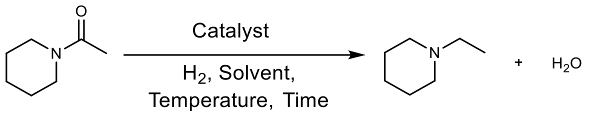

Catalyst	Reaction conditions		Conv. (%)	Select. (%)	Ref.
	Temp. (°C)	P. (bar)			
Pt/Al_2O_3	180	50	16	99	6
Pt/Nb_2O_5	180	50	>99	>99	6
	180	5	58	>99	
Pt/TiO_2	180	50	81	99	6
Pt/ZrO_2	180	50	14	93	6
Pt/SiO_2	180	50	5	80	6
Pt/HBEA	180	50	2	99	6
Pt/HMFI	180	50	2	99	6
Pt/C	180	50	2	99	6
Pt/HAP	70	30	0	0	7
$PtMoOx/TiO_2$	180	50	87	99	6
PtRe/HAP	70	30	0	0	7
PtMo/HAP	70	30	3	99	7
PtV/Al_2O_3	70	30	59	99	7
PtV/SiO_2	70	30	35	99	7
PtV/TiO_2	70	30	41	99	7
PtV/C	70	30	47	99	7
PtV/HAP	70	30	>99	98	7
	70	1[a]	95	>99	
	25	5[a]	88	NA	7
Re/TiO_2	180	50	>99	>99	8
Rh/Re	160	101	>99	96	9
Rh/Mo	160	101	>99	98	9
Ru/Re	160	101	>99	96	9
Rh/Re@C	160	101	>99	98	9

Ru/Re@Al_2O_3	160	101	>99	92	[9]
Rh/Re	160	101	80	85	[10]
Ru/Mo	160	100	>99	90	[11]
Pd/Re/graphite	160	30	>99	>99	[12]
	160	5[b]	90	>99	
2Ru1W/SiO_2	160	50	91	>99	[13]

[a] 40 mol% catalyst, 48 h. [b] 12 mol% catalyst, 20 h.

Table S2. Elemental analysis of ICNPs@Pt/Al_2O_3 by inductively coupled plasma optical emission spectroscopy (ICP-OES).

	**Pt (wt%)**	**Fe (wt%)**	**Al (wt%)**
Theoretical content	0.7	20.0	37.8
Experimental content fresh catalyst	0.6	17.5	30.2
Experimental content after catalysis (5 cycles of 4 h)	0.55	19.7	37.4

Table S3. Summary of the coordination numbers (C.N.), path distances (R, in Å), and Debye–Waller factor (σ^2) of Pt–Pt and Pt–O in fresh/spent ICNPs@Pt/Al_2O_3 catalysts in comparison with Pt foil, determined by EXAFS fitting.

**Sample**	**Path**	**C.N.**	**R (Å)**	**□^{2} (Å^2)**	**E_0 (eV)**
Pt foil	Pt–Pt	12 (fixed)	2.76± 0.01	0.004± 0.0002	11571.5 ± 0.3
ICNPs@Pt/Al_2O_3	Pt–Pt	8.2± 0.7	2.78± 0.01	0.006± 0.0005	11570.3 ± 0.7
	Pt–O	1.0± 0.2	1.96± 0.01	0.006± 0.0005	11570.3 ± 0.7
ICNPs@Pt/Al_2O_3 after 4 cycles	Pt–Pt	7.3± 0.7	2.74± 0.01	0.006± 0.0006	11570.3 ± 0.9
	Pt–O	1.4± 0.2	1.96± 0.01	0.006± 0.0006	11570.3 ± 0.9

Table S4. Experimental estimation of the surface temperature of ICNPs@Pt/Al_2O_3 heated by magnetic induction.

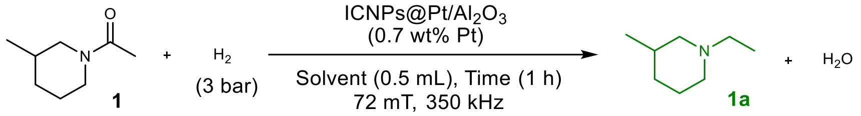

Solvent	b.p. (°C)	Boiling?	$T_{Reactor}$ (°C)	Conv. (%)	Y_{1a} (%)
Dimethoxyethane	85	Yes	85	0	0
Heptane	98	Yes	90	0	0
Dioxane	101	Yes	95	2	2
p-Xylene	139	Yes	130	2	2
Mesitylene	165	Yes	150	70	70
Decalin	186	Yes	162	77	77
Dodecane	216	Yes	150	71	71
Propylene carbonate	242	Yes	151	0[b]	0[b]
Tetradecane	254	Yes	n.d.	n.d.	n.d.
Hexadecane	287	Yes	205	>99	54[c]
Tetraethylene glycol	327	No	n.d.	n.d.	n.d.

Local temperature at solvent/ICNPs@Pt/Al_2O_3 interface estimated from local boiling/gas bubble formation of respective solvent. Experimental conditions: ICNPs@Pt/Al_2O_3 (35.0 mg), solvent (0.5 mL), $Ar_{(g)}$, magnetic field (μ_0H_{max} = 72 mT, 350 kHz), 5 min. For catalysis: **1** (12.9 mg, 0.1 mmol), ICNPs@Pt/Al_2O_3 (35.0 mg, 1.26 μmol Pt), magnetic field (μ_0H_{max} = 72 mT, 350 kHz), solvent (0.5 mL), H_2 (3 bar), 1 h. Products yields determined by GC-FID using tetradecane as the internal standard. [a] Determined using an infrared camera. [b] Severe solvent decomposition. [c] Low selectivity due to C-N bond cleavage. T. = temperature. Conv. = conversion. Y_{1a} = yield of **1a**. n.d. = no data.

Table S5. Recycling experiments with ICNPs@Pt/Al_2O_3 prepared without heat treatment to anchor the ICNPs.

Entry	Cycle	Conversion (%)	Yield 1a (%)
1	1	95	95
2	2	97	97
3	3	65	65

Reaction condition: **1** (12.9 mg, 0.1 mmol), ICNPs@Pt/Al_2O_3 without heat treatment (35.0 mg, 1.26 μmol Pt), magnetic field (μ_0H_{max} = 72 mT, 350 kHz), decalin (0.5 mL), H_2 (3 bar), 2 h. Products yield determined by GC-FID using tetradecane as the internal standard.

Table S6. XRF analysis of reaction solutions after each cycle of 4 h under standard conditions (cf. Figure S8).

Reaction cycle	Fe content [ppm]	Pt content [ppm]
1	<0.3	<0.3
2	<0.3	<0.3
3	<0.3	<0.3
4	<0.3	<0.3
5	<0.3	<0.3

Supplementary Figures

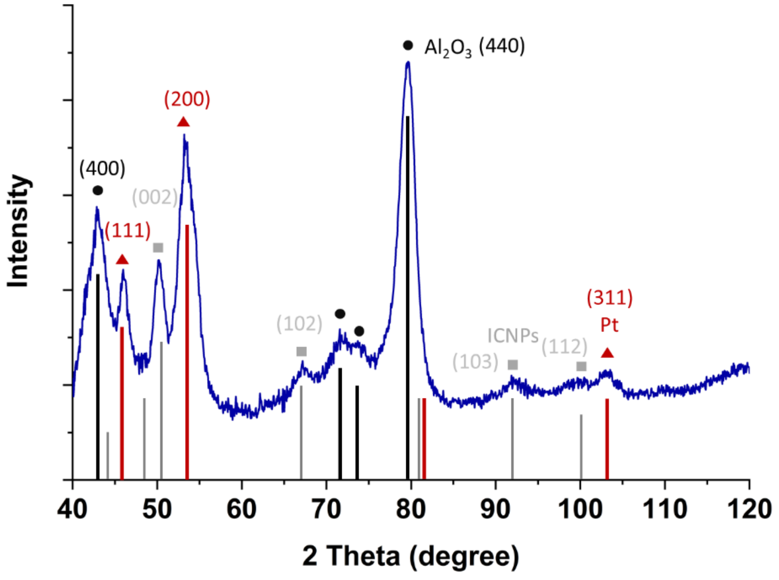

Figure S1. Powder XRD diffractogram of ICNPs@Pt/Al_2O_3. References: γ-Al_2O_3 (black dot), *fcc* Pt(0) (red triangle) and ICNPs (grey square).

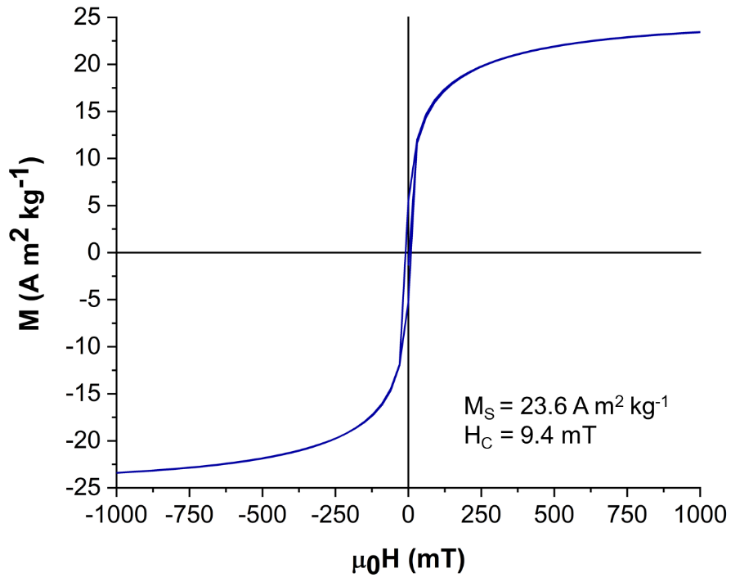

Figure S2. Superconducting quantum interference device (SQUID) measurement at 300 K. The saturation magnetization (M_S) of per unit weight of Fe is 168.5 A m^2 kg^{-1}, consistent with previously reported values.

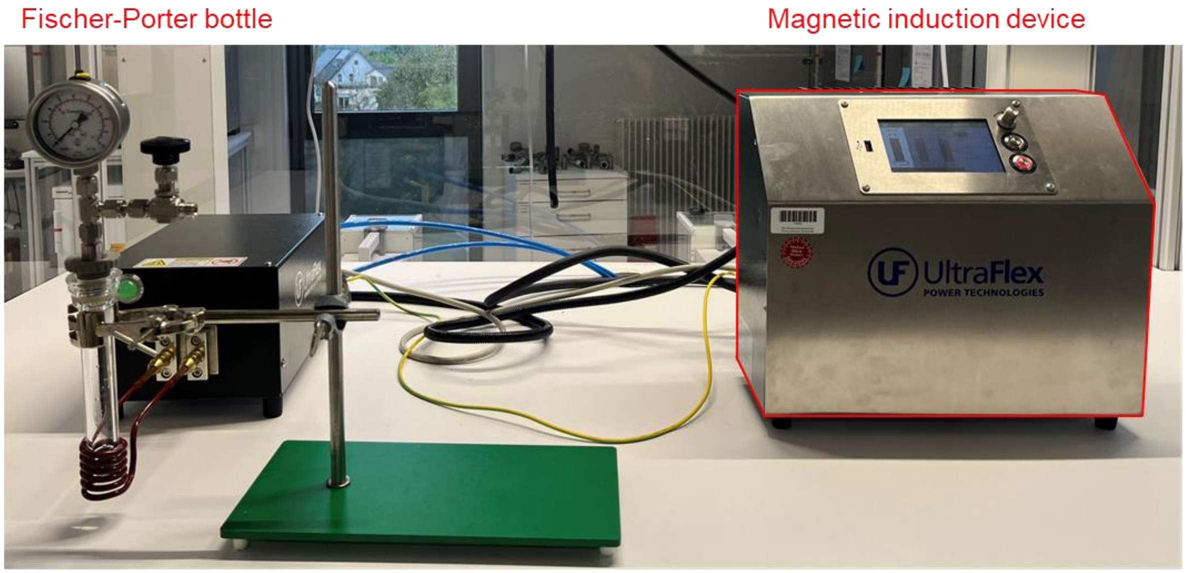

Figure S3. Magnetic induction reaction setup.

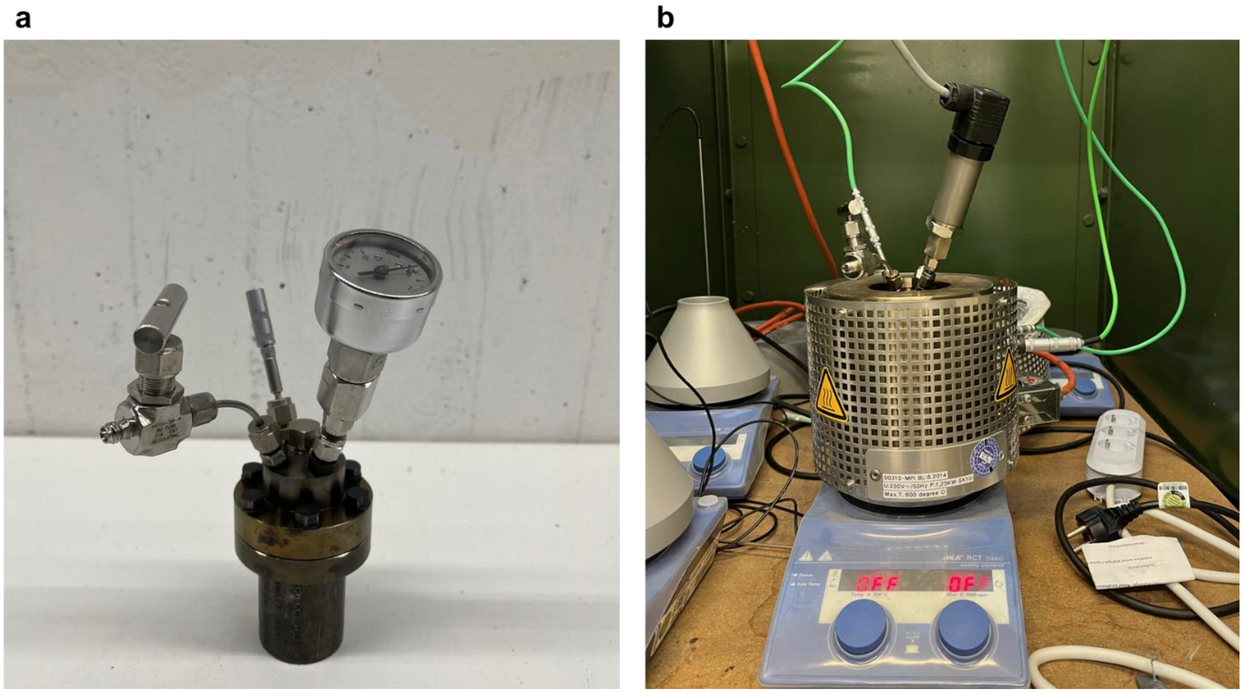

Figure S4. Autoclave reaction setup. a) Autoclave. b) Autoclave reactor in a heating bag.

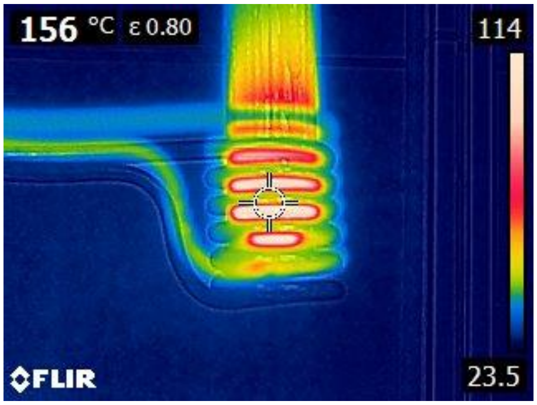

Figure S5. The global temperature of the reactor surface resulting from the heat dissipated by the ICNPs@Pt/Al_2O_3 was measured using an infrared camera under standard conditions (72 mT, 350 kHz).

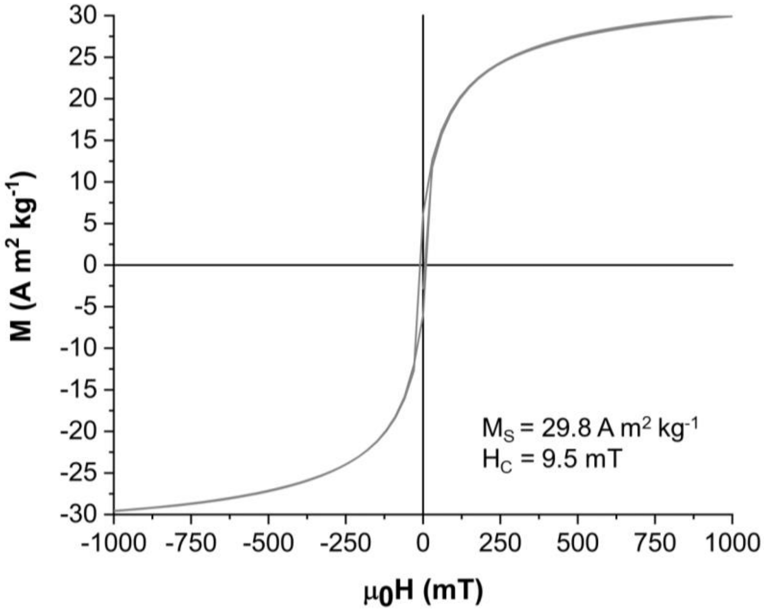

Figure S6. Superconducting quantum interference device (SQUID) measurement after catalysis at 300 K.

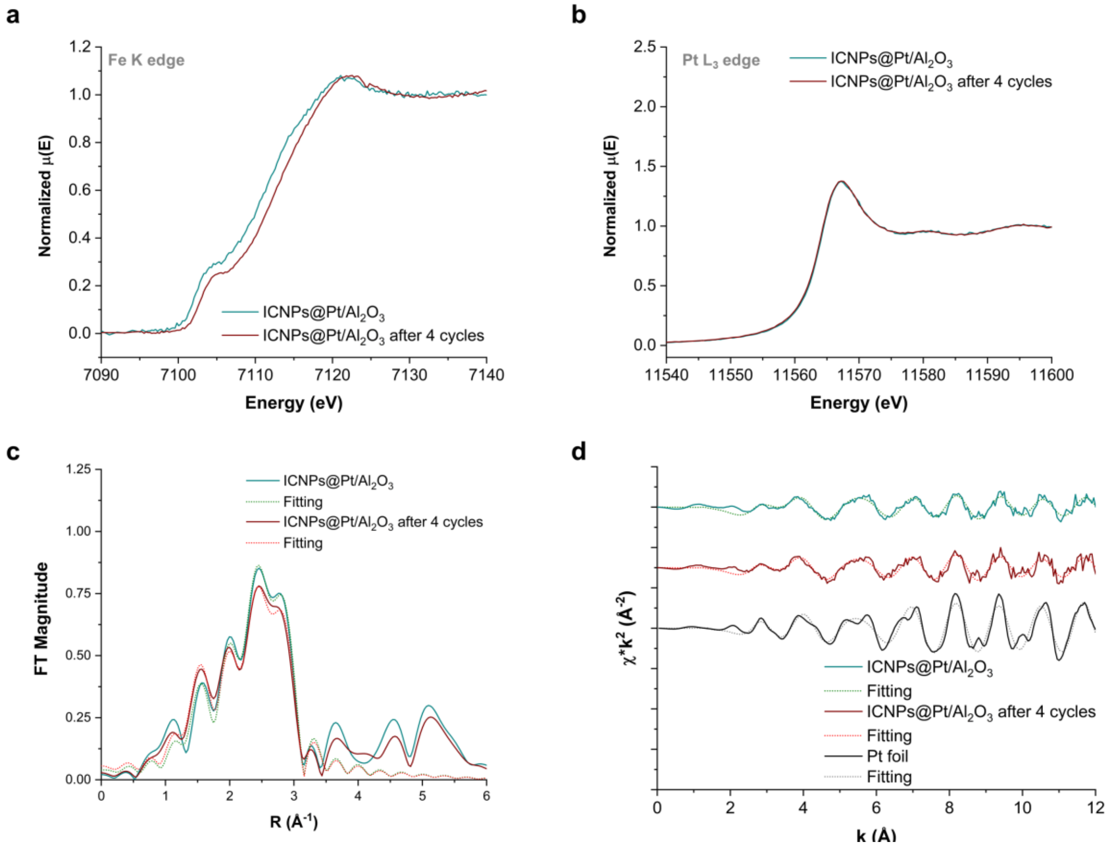

Figure S7. The XANES and EXAFS measurement. a) Fe K-edge XANES spectra (normalized), b) Pt L_3-edge XANES spectra (normalized), c) Fourier transform magnitudes of Pt L_3-edge k^2-weighted EXAFS spectra in R-space and d) Pt L_3-edge k^2-weighted EXAFS in k-space.

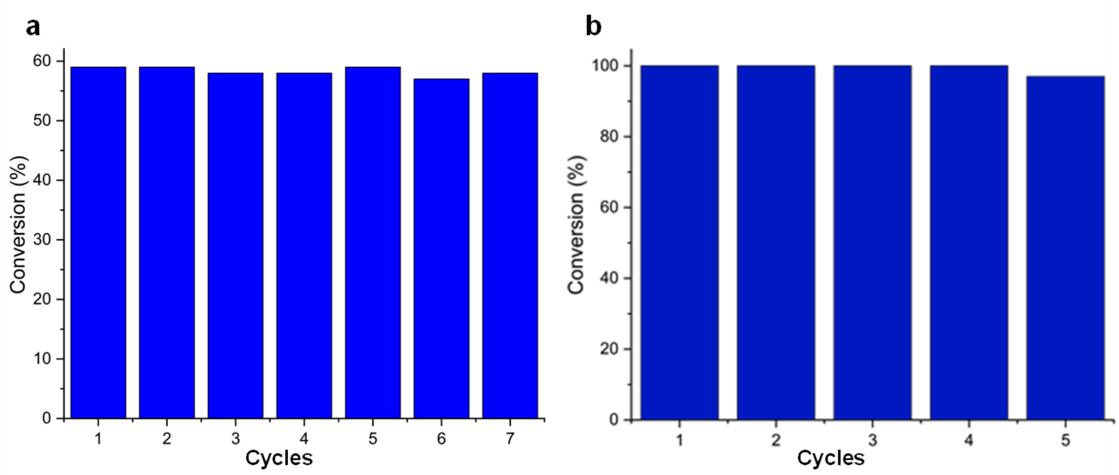

Figure S8. Study of the stability of ICNPs@Pt/Al_2O_3 through recycling experiments for the conversion of **1** to **1a** at a) incomplete and b) complete conversion. Reaction conditions: **1** (25.8 mg, 0.2 mmol in **a**; 12.9 mg, 0.1 mmol in **b**), ICNPs@Pt/Al_2O_3 (35.0 mg, 1.26 µmol Pt), decalin (0.5 mL), H_2 (3 bar), 4 h, magnetic field ($\mu_0 H_{max}$ = 72 mT, 350 kHz). Products yields determined by GC-FID using tetradecane as the internal standard. The product selectivity to **1a** is >99%.

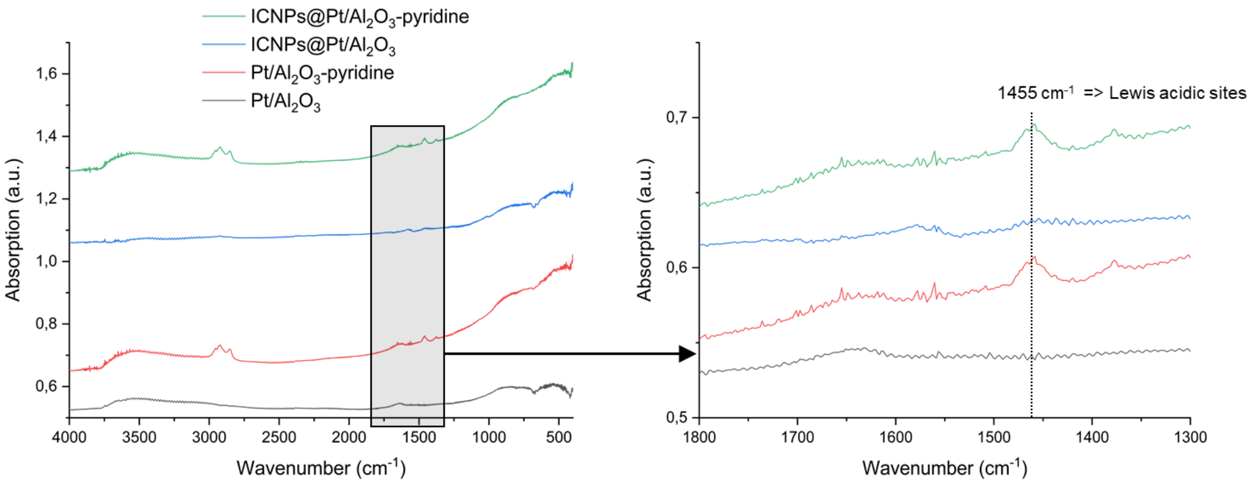

Figure S9. Transmission IR of Pt/Al_2O_3 and ICNPs@Pt/Al_2O_3 before and after adsorption of pyridine as a molecular probe.

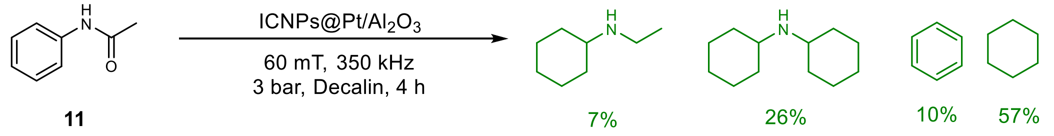

Figure S10. Hydrogenation of **11** using ICNPs@Pt/Al_2O_3 at 60 mT for 4 h. Reaction conditions: **11** (12.9 mg, 0.10 mmol), ICNPs@Pt/Al_2O_3 (35.0 mg, 1.26 µmol Pt), decalin (0.5 mL), H_2 (3 bar).

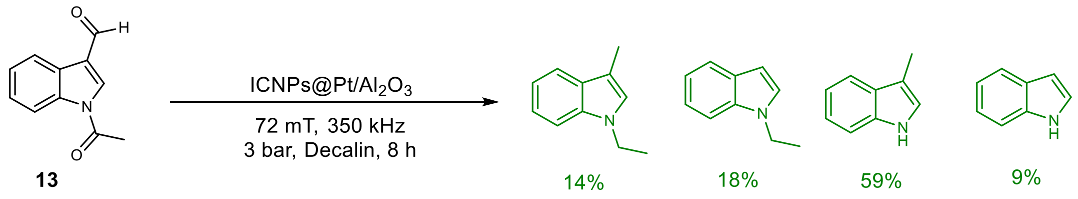

Figure S11. Hydrogenation of **12** using ICNPs@Pt/Al_2O_3 at 70 mT for 8 h. Reaction conditions: **13** (18.7 mg, 0.10 mmol), ICNPs@Pt/Al_2O_3 (35.0 mg, 1.26 µmol Pt), decalin (0.5 mL), H_2 (3 bar).

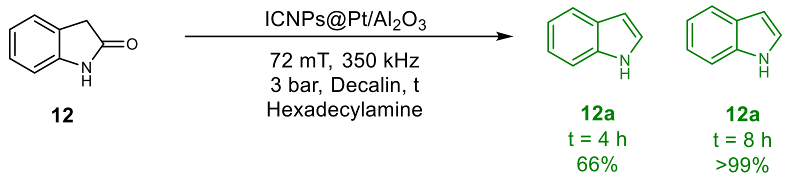

Figure S12. Hydrogenation of **13** using ICNPs@Pt/Al_2O_3 at 70 mT for 4 h in the presence of hexadecylamine as an additive. Reaction conditions: **12** (13.3 mg, 0.10 mmol), ICNPs@Pt/Al_2O_3 (35.0 mg, 1.26 µmol Pt), hexadecylamine (20 mg, 0.08 mmol), decalin (0.5 mL), H_2 (3 bar). >99% selectivity to **12a**.

Isolated Yields

General procedure: ICNPs@Pt/Al_2O_3 (35.0 mg) and substrate (0.40 mmol) were dispersed in decalin (0.5 mL) in a Fisher-Porter bottle inside the glovebox, then sealed and pressurized with H_2 (3 bar). The Fisher-Porter bottle was placed in the coil under an alternating magnetic field for selected of the time. After the reaction, the catalyst was removed by magnetic separation and filtration and 1.0 M HCl in diethyl ether was added to the resulting solution, immediately producing the precipitant. The hydrochloride salt was then filtered and dried. The desired product was isolated as a solid.

<u>Hydrogenation of 1-acetyl-3-methyl piperidine (**1**)</u>

^{1}H NMR (400 MHz, $CDCl_3$) *δ* (ppm): δ = 11.77 (br, 1H), 3.53 (d, 2H), 3.38 (d, 2H), 3.04 (br, 2H), 2.49-2.14 (m, 4H), 1.91-1.82 (t, 2H), 1.46-1.43 (t, 3H), 1.09-1.04 (m, 1H), 1.01 (t, 3H). ^{13}C NMR (100 MHz, $CDCl_3$) δ = 58.46, 52.65, 52.26, 22.60, 18.98, 9.19.

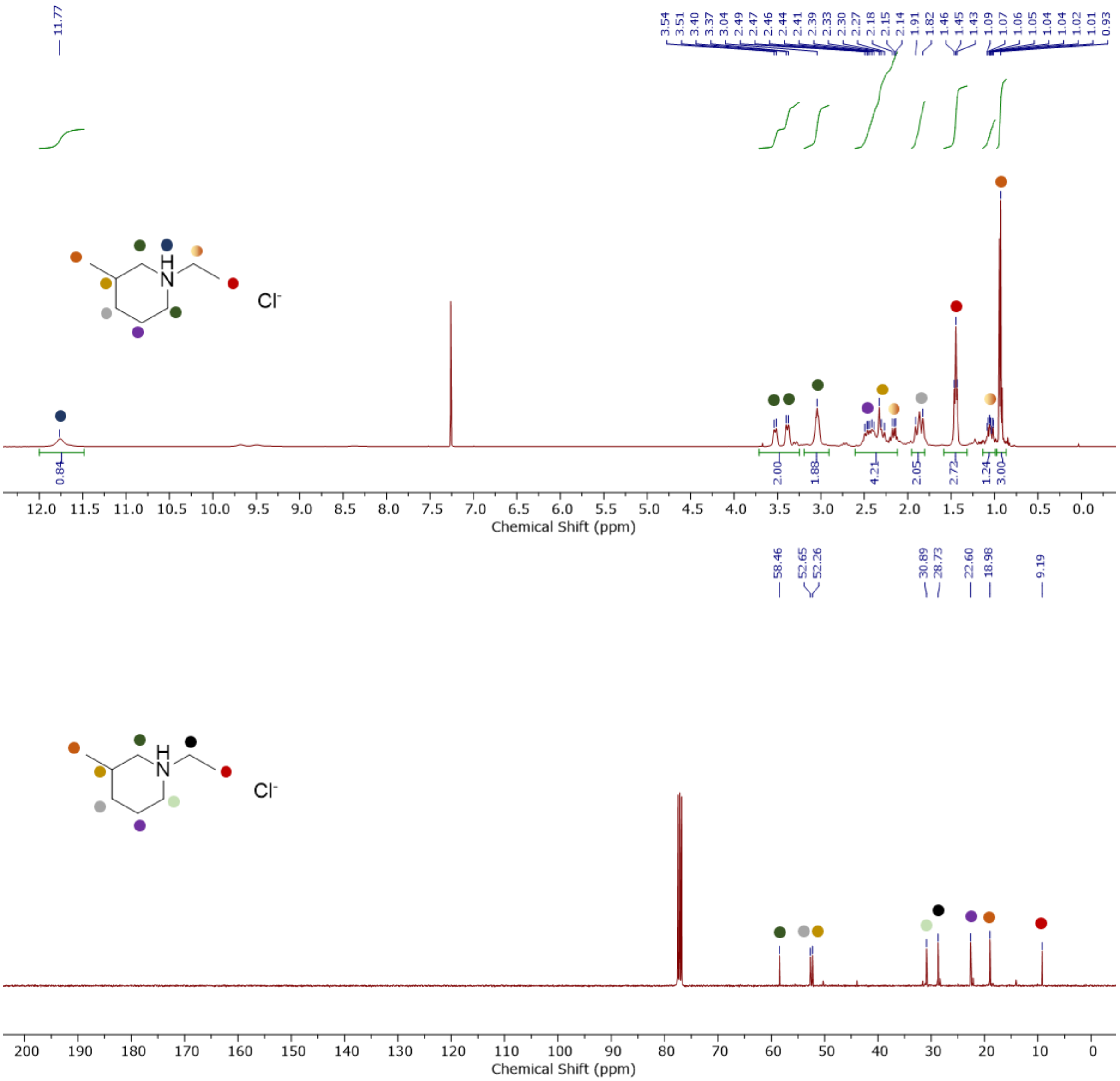

Figure S13. ^{1}H and ^{13}C NMR spectra of isolated **1a**.

Hydrogenation of 1-methyl-2-piperidone (**6**)

^{1}H NMR (400 MHz, $CDCl_3$) δ (ppm): δ = 11.68 (br, 1H), 3.43 (d, 2H), 2.73 (d, 3H), 2.70-2.66 (m, 2H), 2.19-2.14 (m, 2H), 1.90-1.79 (m, 3H), 1.41-1.33 (m, 1H). ^{13}C NMR (100 MHz, $CDCl_3$) δ = 55.06, 43.96, 22.80, 21.43.

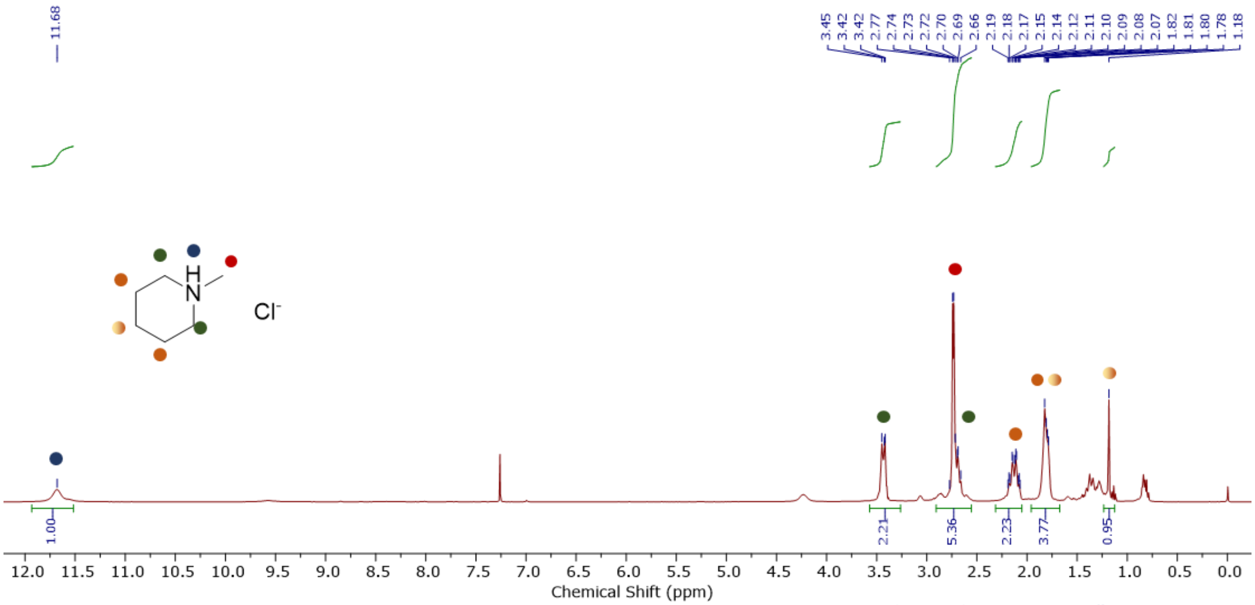

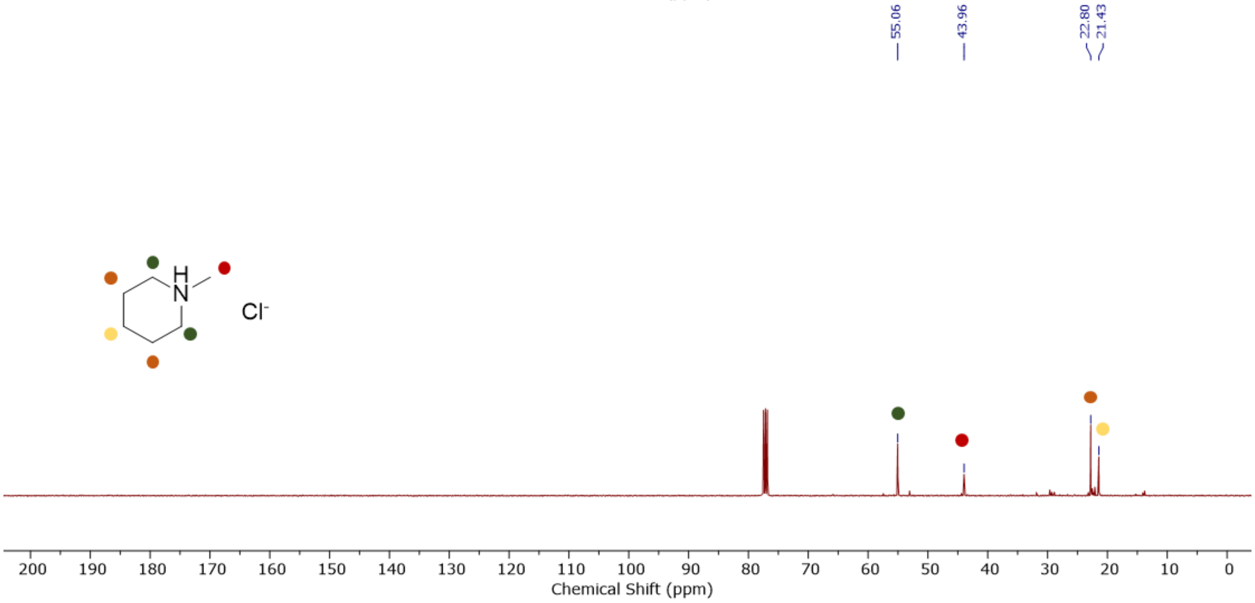

Figure S14. ^{1}H and ^{13}C NMR spectra of isolated **6a**.

GC-FID Chromatograms of crude mixtures

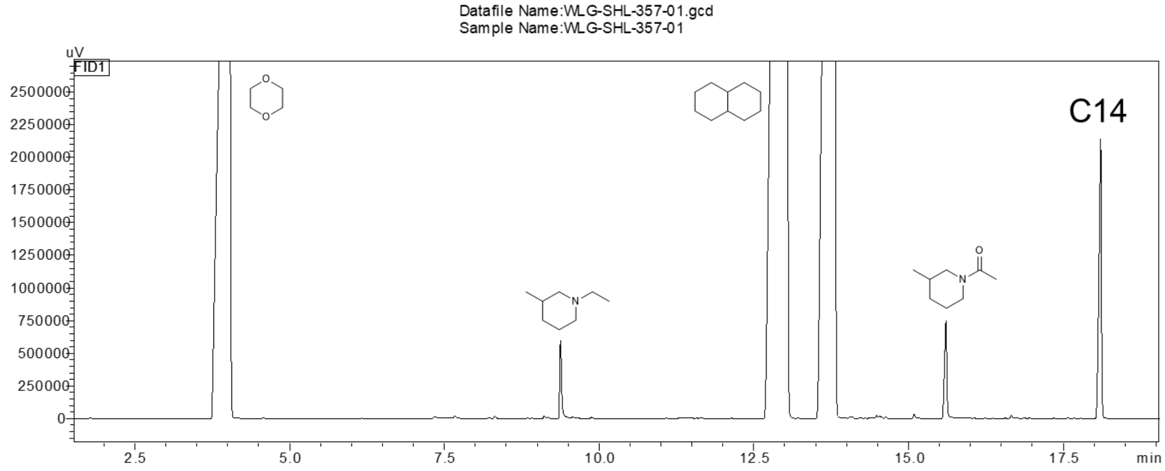

Figure S15. Chromatogram corresponding to the data of Table 1, Entry 1.

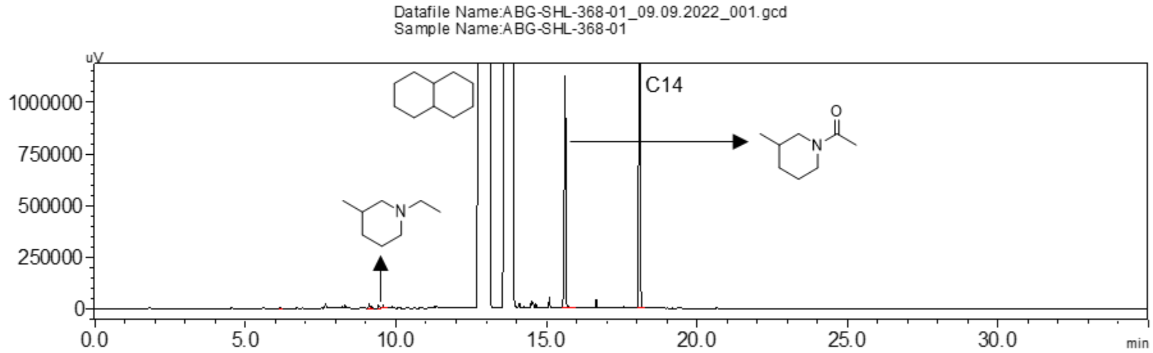

Figure S16. Chromatogram corresponding to the data of Table 1, Entry 2.

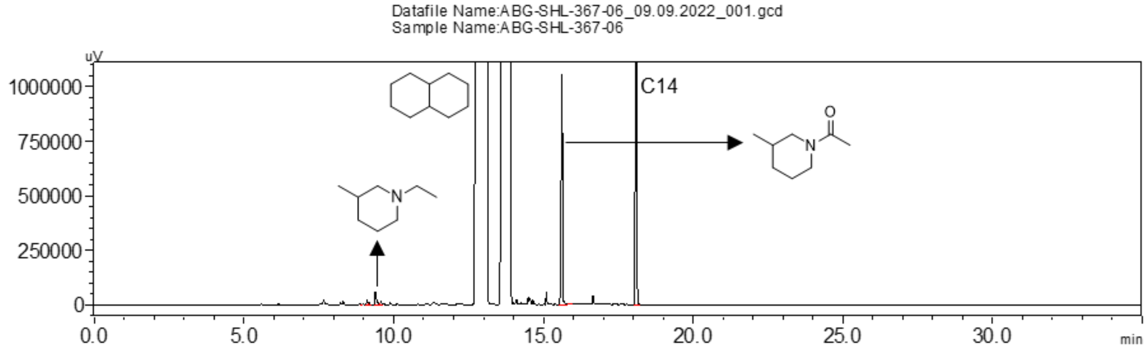

Figure S17. Chromatogram corresponding to the data of Table 1, Entry 3.

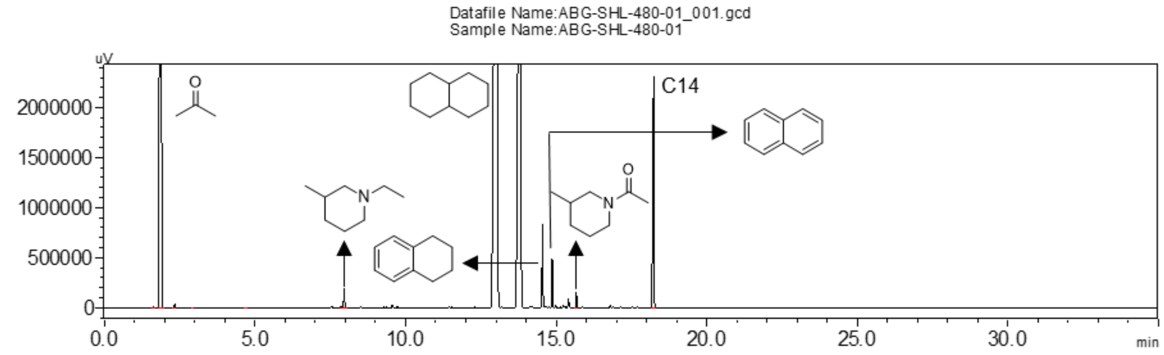

Figure S18. Chromatogram corresponding to the data of Table 1, Entry 4 (low mass balance).

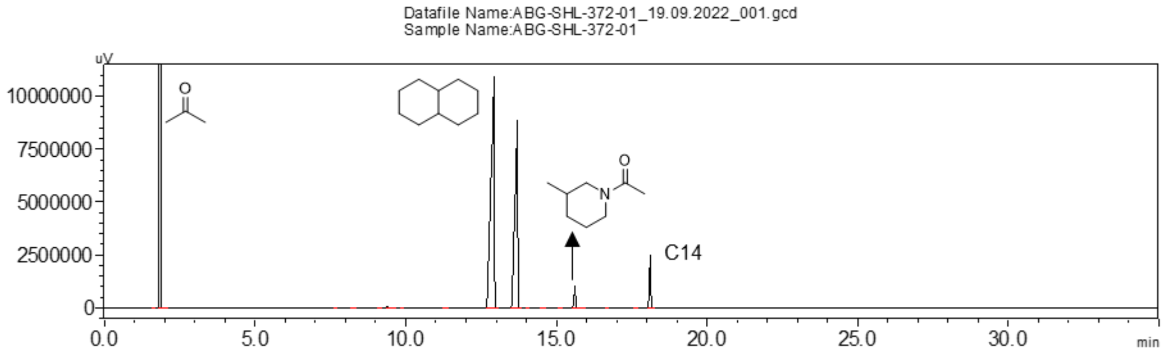

Figure S19. Chromatogram corresponding to the data of Table 1, Entry 5.

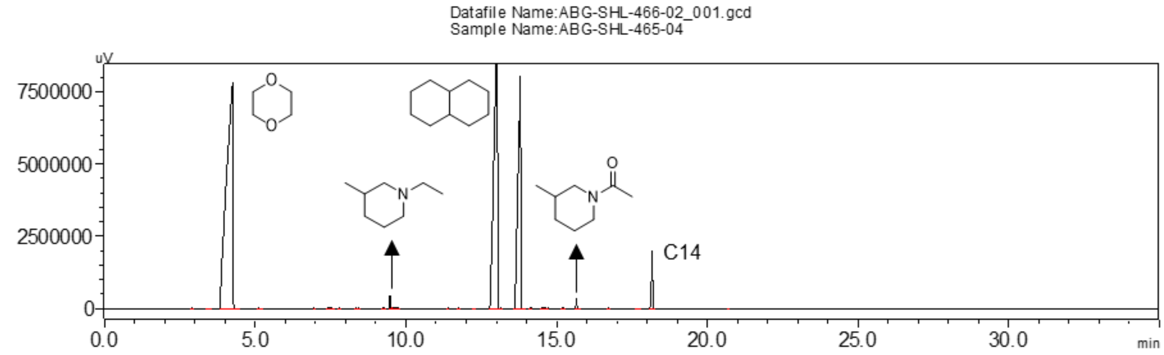

Figure S20. Chromatogram corresponding to the data of Table 1, Entry 6.

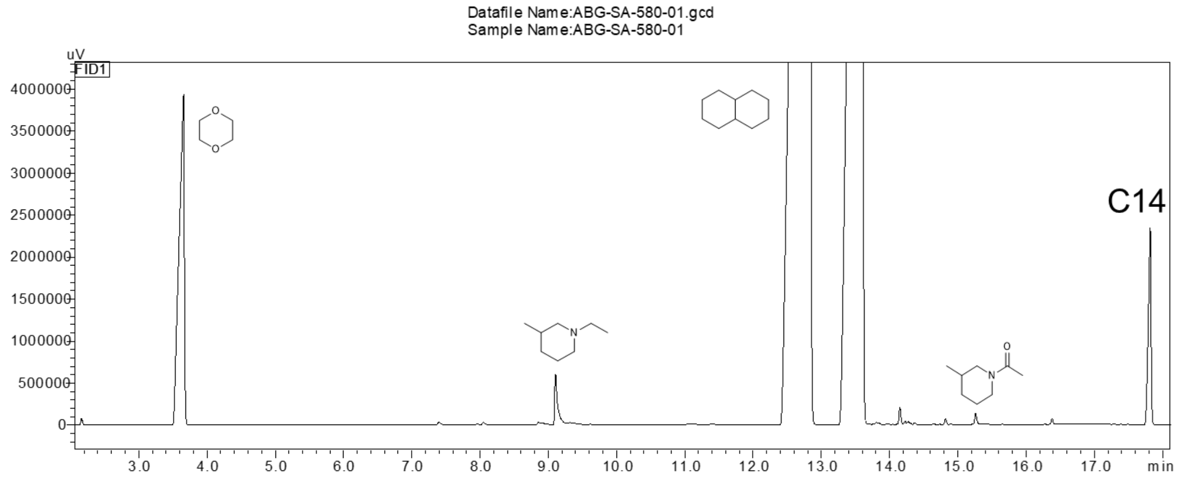

Figure S21. Chromatogram corresponding to the data of Table 1, Entry 7.

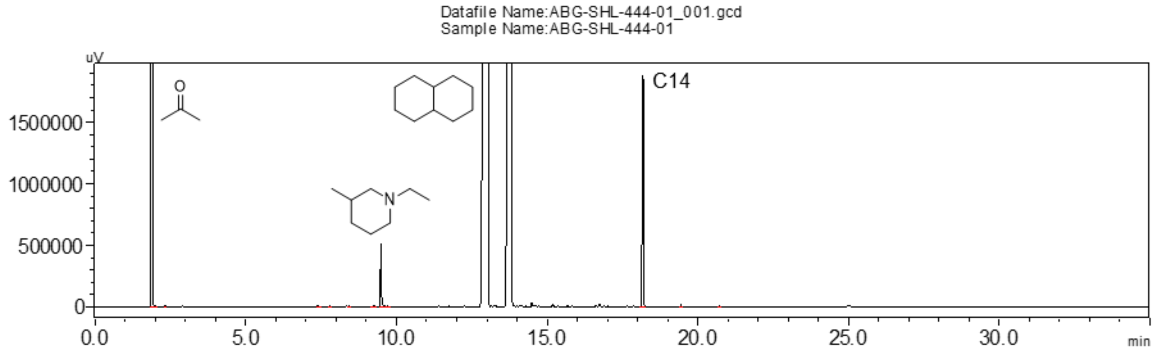

Figure S22. Chromatogram corresponding to the data of Table 1, Entry 8.

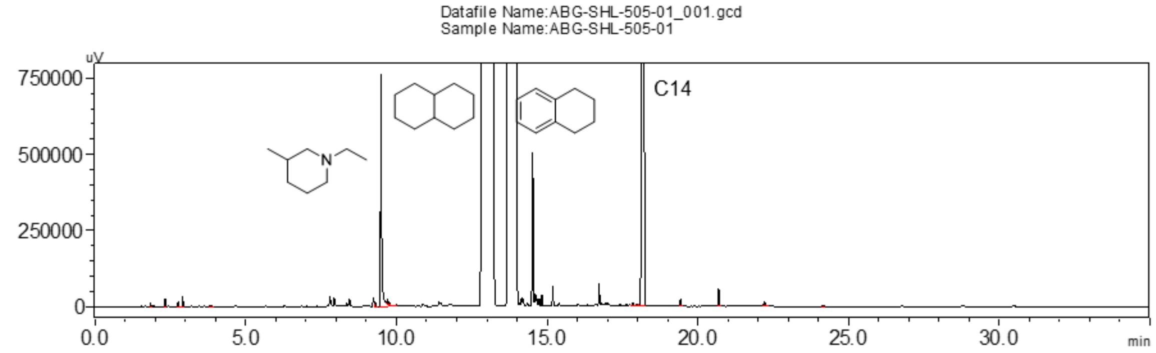

Figure S23. Chromatogram corresponding to the data of Table 1, Entry 9.

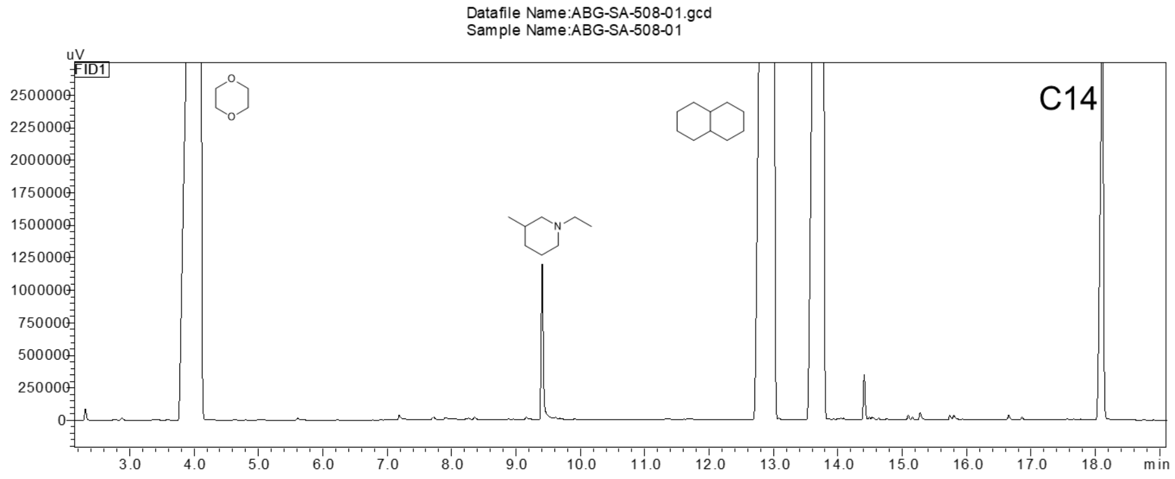

Figure S24. Chromatogram corresponding to the data of Table 1, Entry 10.

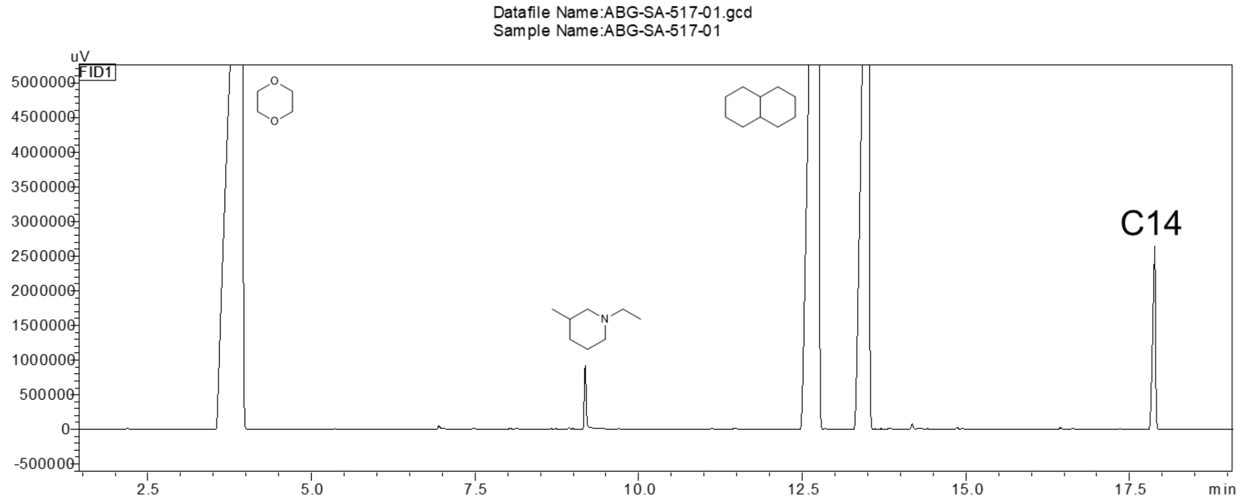

Figure S25. Chromatogram corresponding to the data of Table 1, Entry 11.

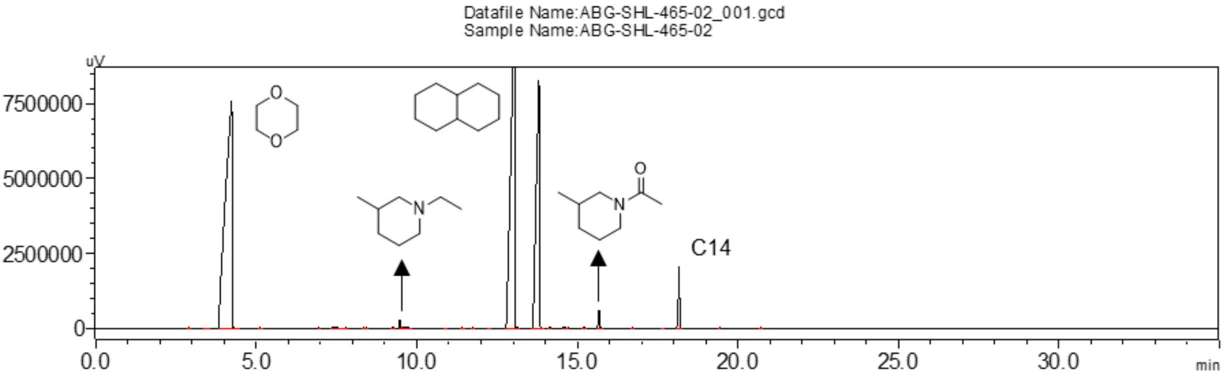

Figure S26. Chromatogram corresponding to the data of Table 1, Entry 12.

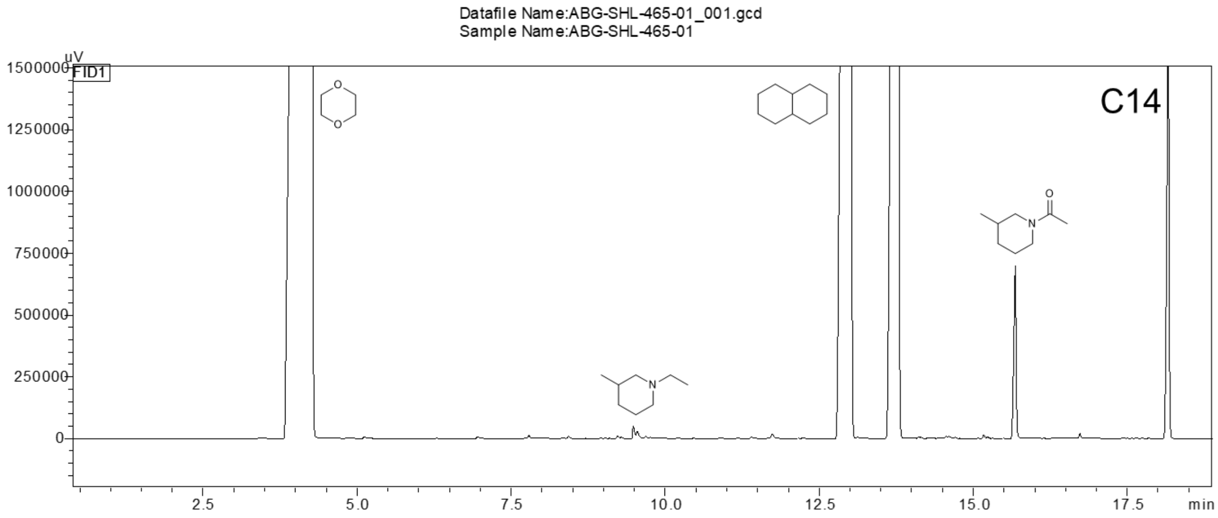

Figure S27. Chromatogram corresponding to the data of Table 1, Entry 13.

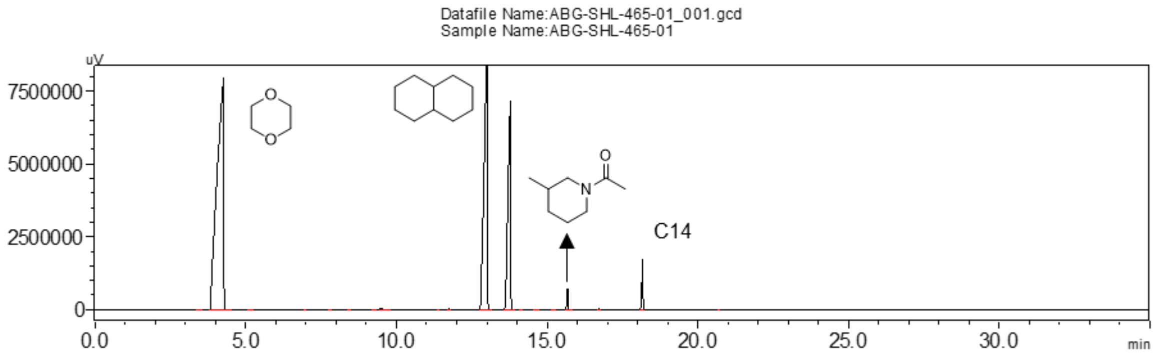

Figure S28. Chromatogram corresponding to the data of Table 1, Entry 14.

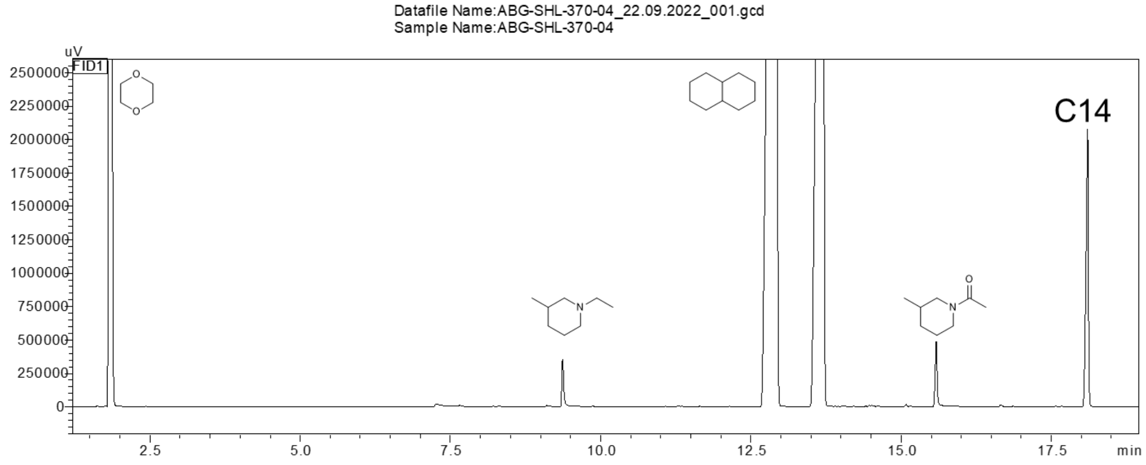

Figure S29. Chromatogram corresponding to the data of Table 1, Entry 15.

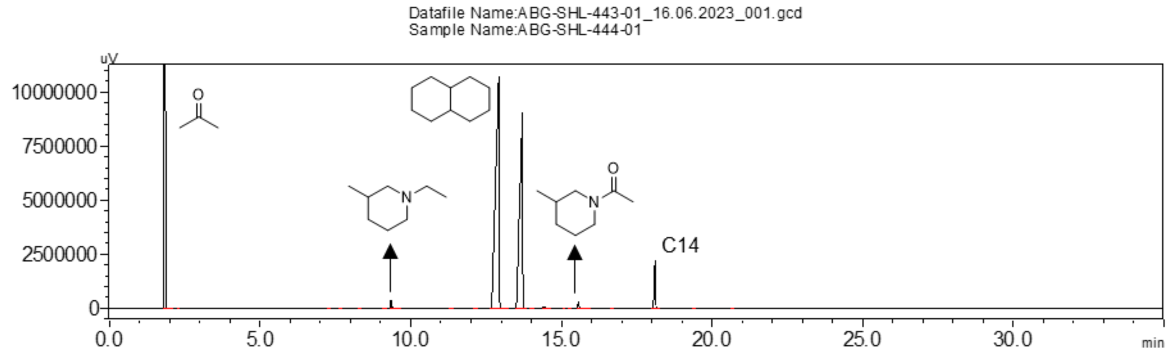

Figure S30. Chromatogram corresponding to the data of Table 1, Entry 16.

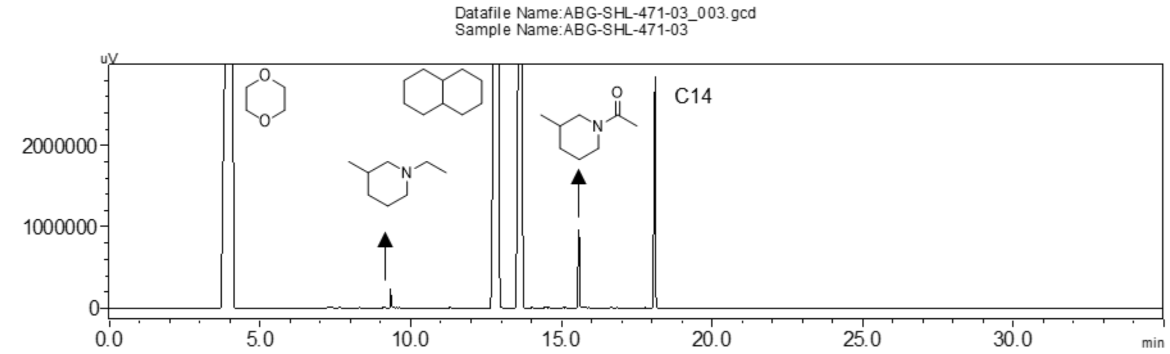

Figure S31. Chromatogram corresponding to the data of Figure 4, 0.5 h.

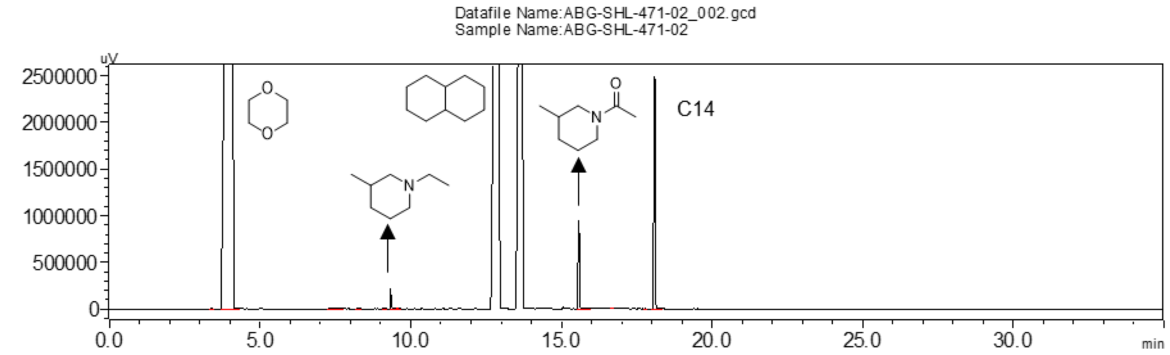

Figure S32. Chromatogram corresponding to the data of Figure 4, 0.5 h **(switch off).**

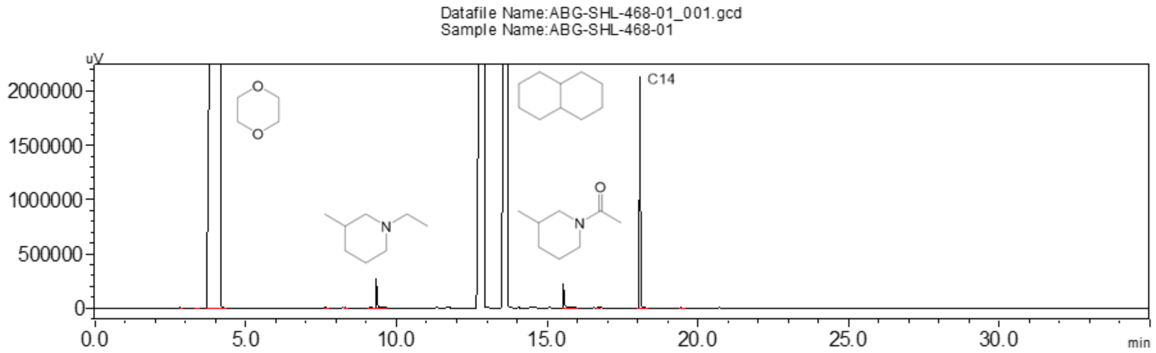

Figure S33. Chromatogram corresponding to the data of Figure 4, 1 h.

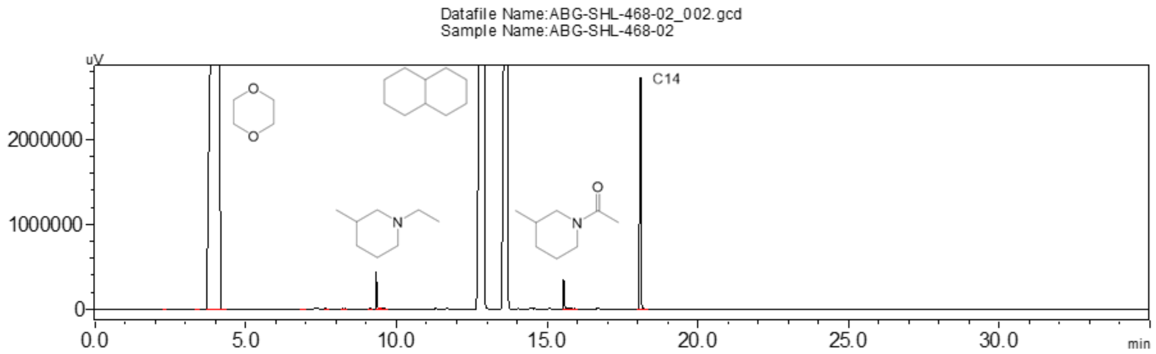

Figure S34. Chromatogram corresponding to the data of Figure 4, 1 h **(switch off).**

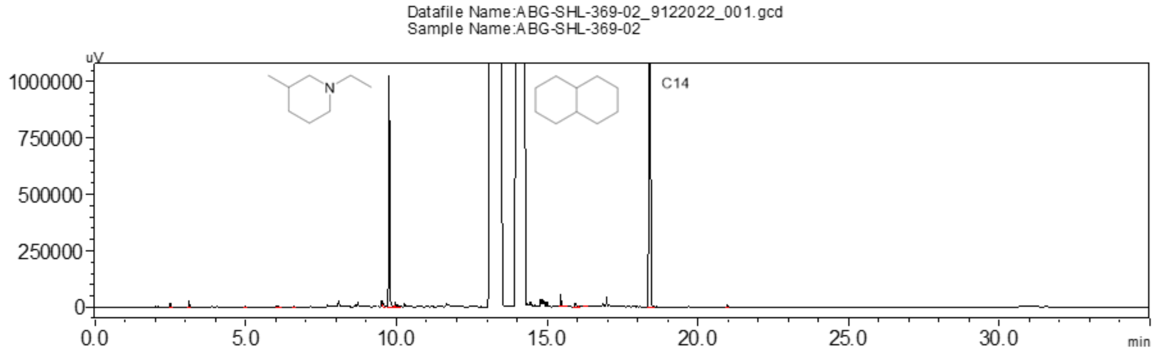

Figure S35. Chromatogram corresponding to the data of Figure 4, 2 h.

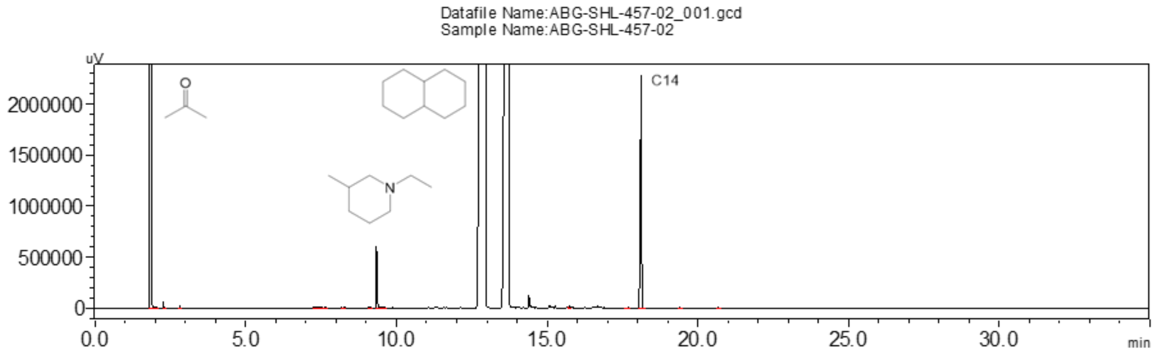

Figure S36. Chromatogram corresponding to the data of Figure 4, 2 h **(switch off).**

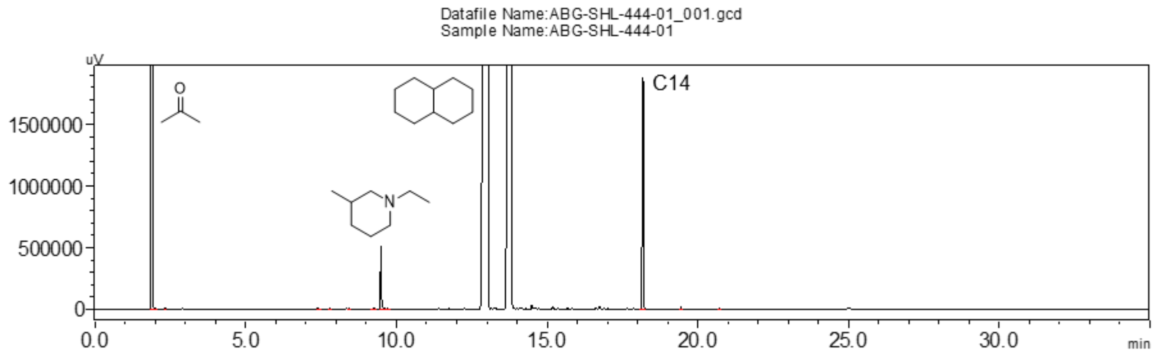

Figure S37. Chromatogram corresponding to the data of Figure 4, 4 h.

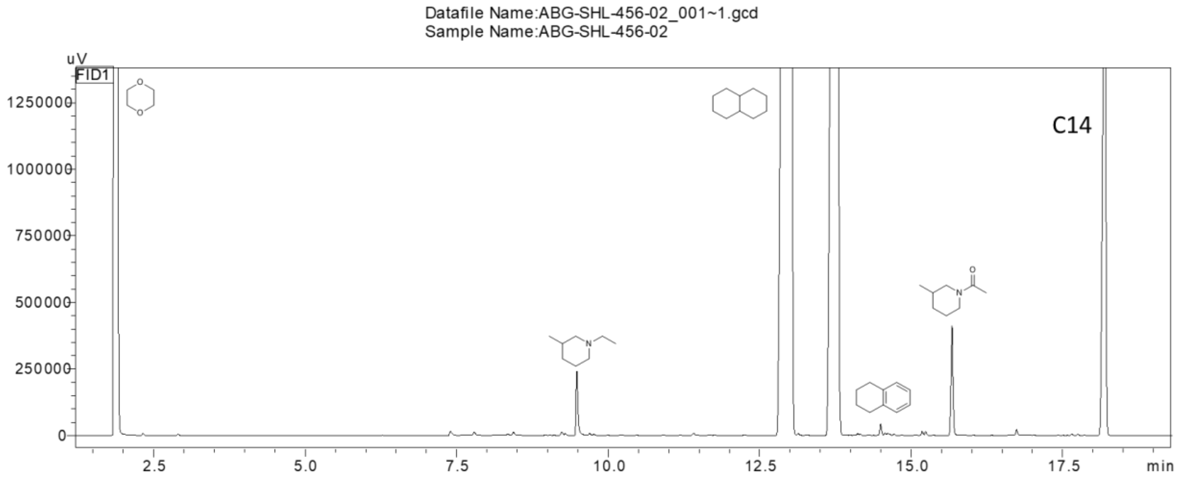

Figure S38. Chromatogram corresponding to the data of Figure 5h, cycle 1.

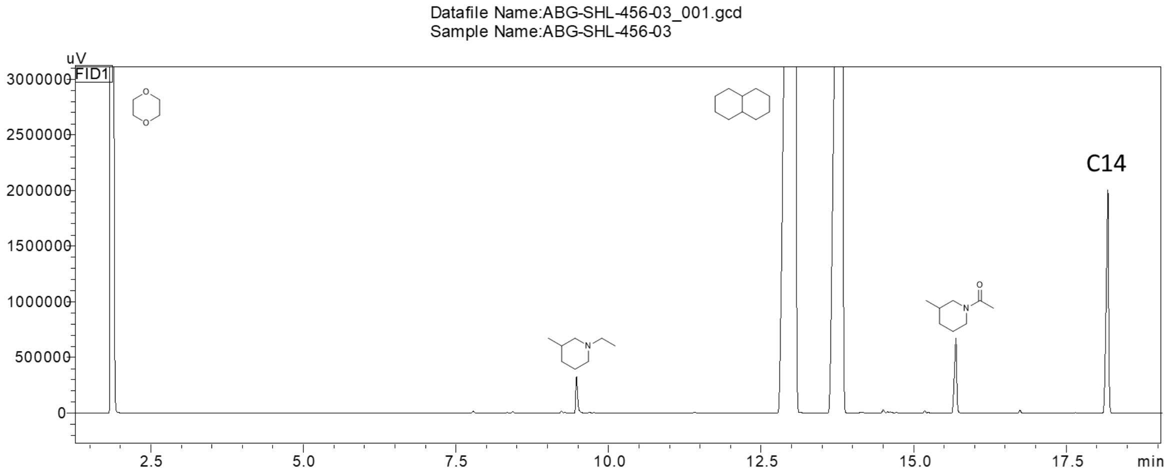

Figure S39. Chromatogram corresponding to the data of Figure 5h, cycle 2.

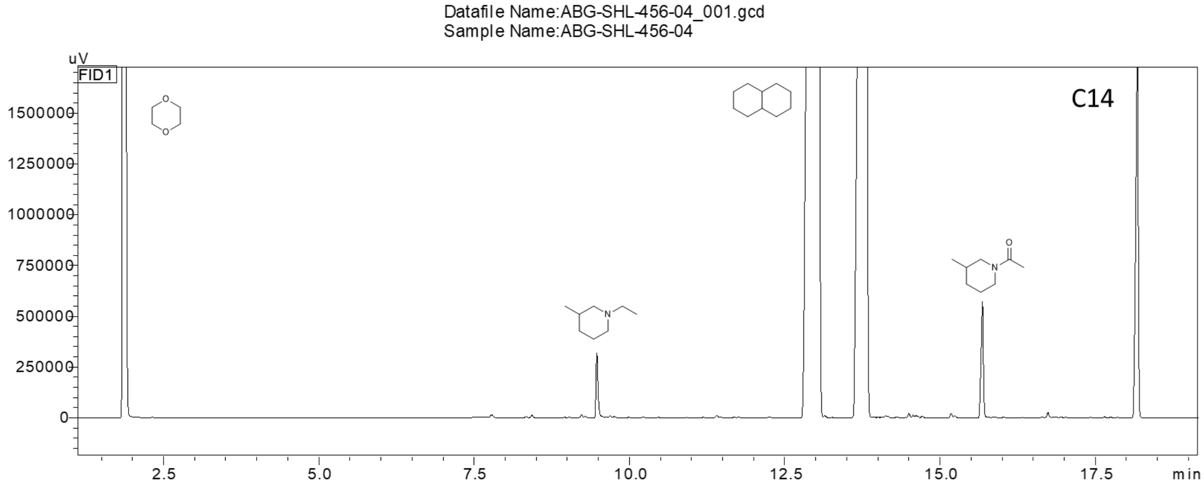

Figure S40. Chromatogram corresponding to the data of Figure 5h, cycle 3.

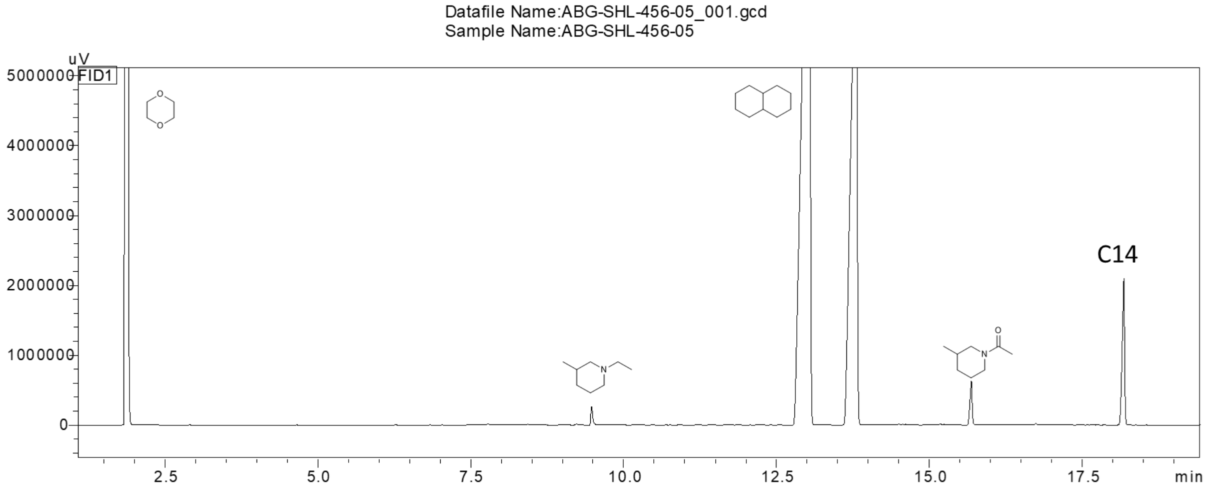

Figure S41. Chromatogram corresponding to the data of Figure 5h, cycle 4.

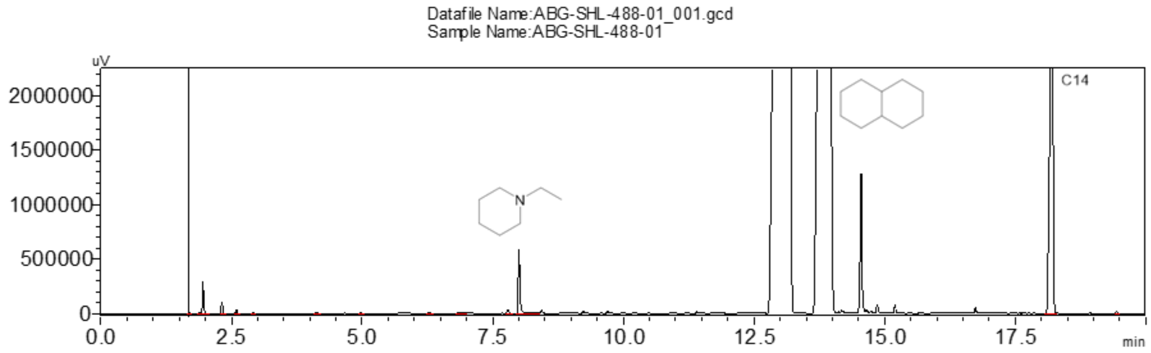

Figure S42. Chromatogram corresponding to Table 2, Substrate **2**.

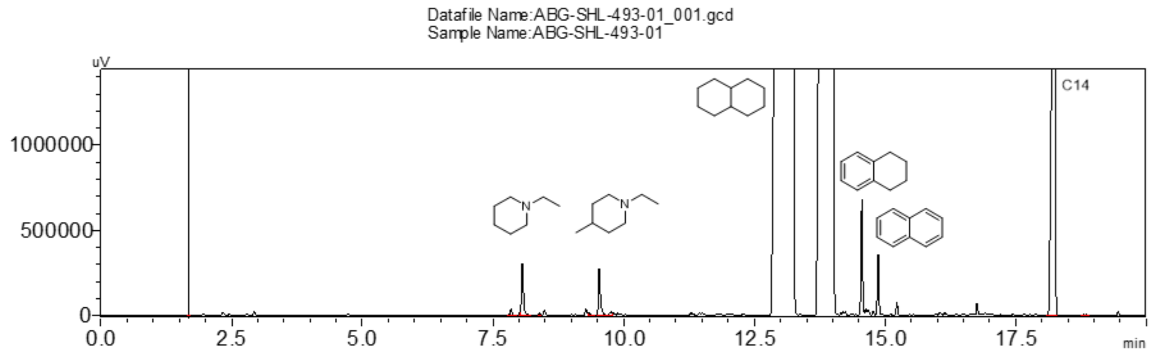

Figure S43. Chromatogram corresponding to Table 2, Substrate **3**.

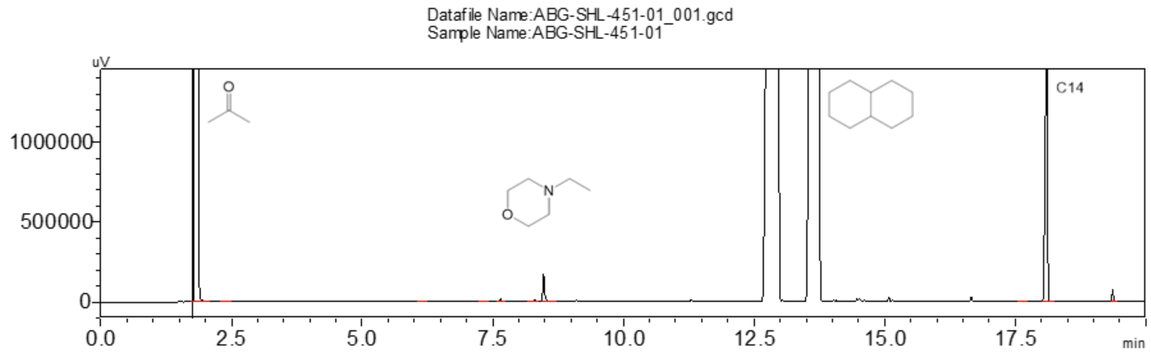

Figure S44. Chromatogram corresponding to Table 2, Substrate **4**.

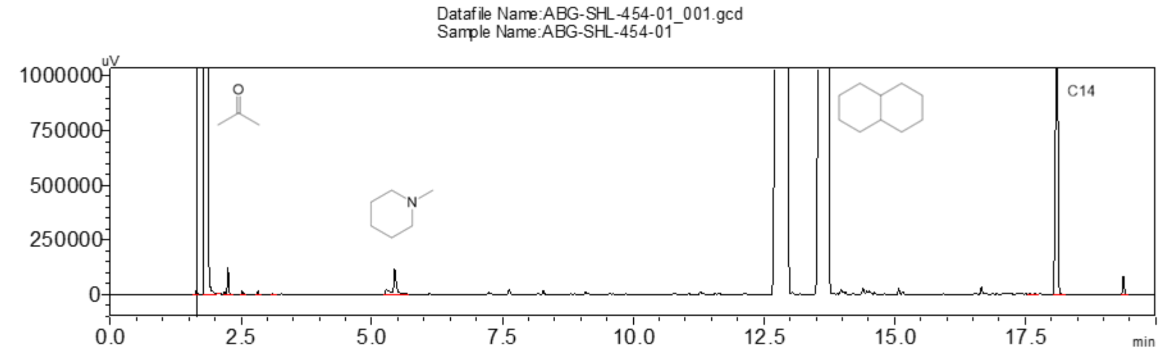

Figure S45. Chromatogram corresponding to Table 2, Substrate **5**.

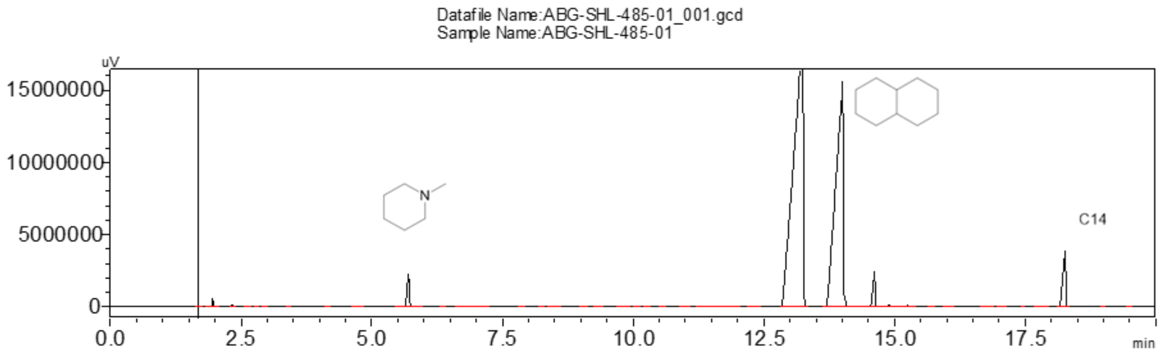

Figure S46. Chromatogram corresponding to Table 2, Substrate **6**.

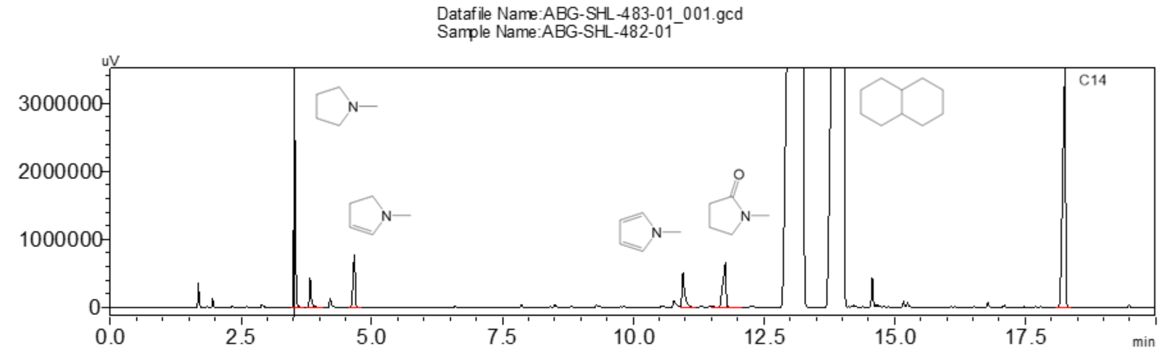

Figure S47. Chromatogram corresponding to Table 2**,** Substrate **7**.

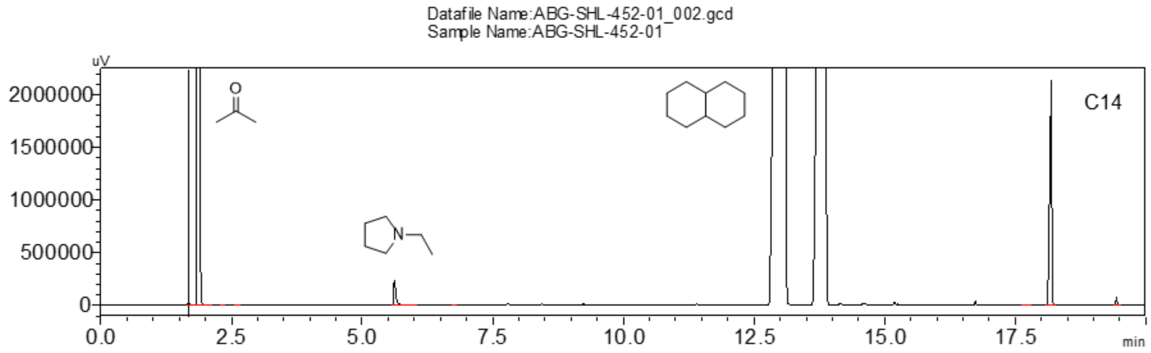

Figure S48. Chromatogram corresponding to Table 2**,** Substrate **8**.

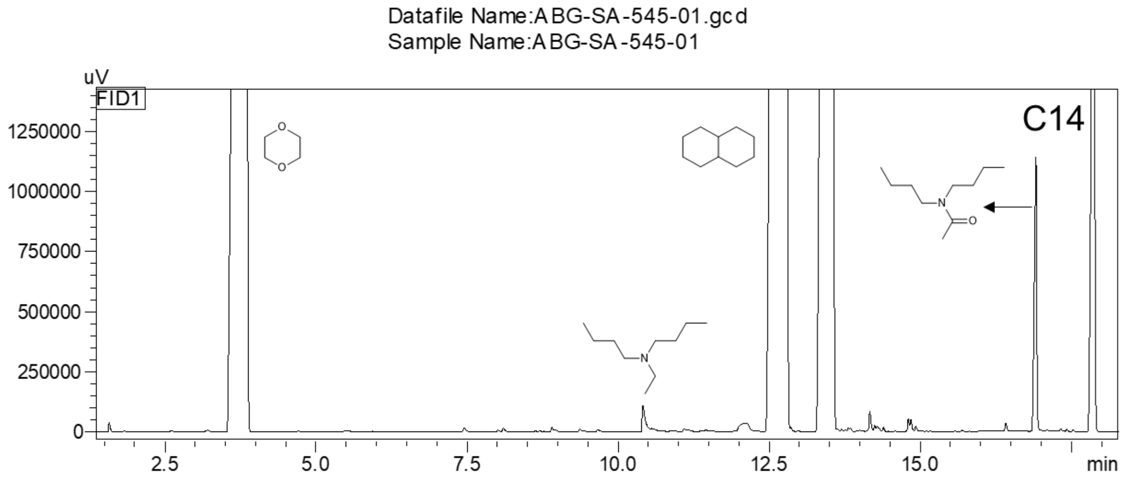

Figure S49. Chromatogram corresponding to the data of Table 2, Substrate **9**, 2 h.

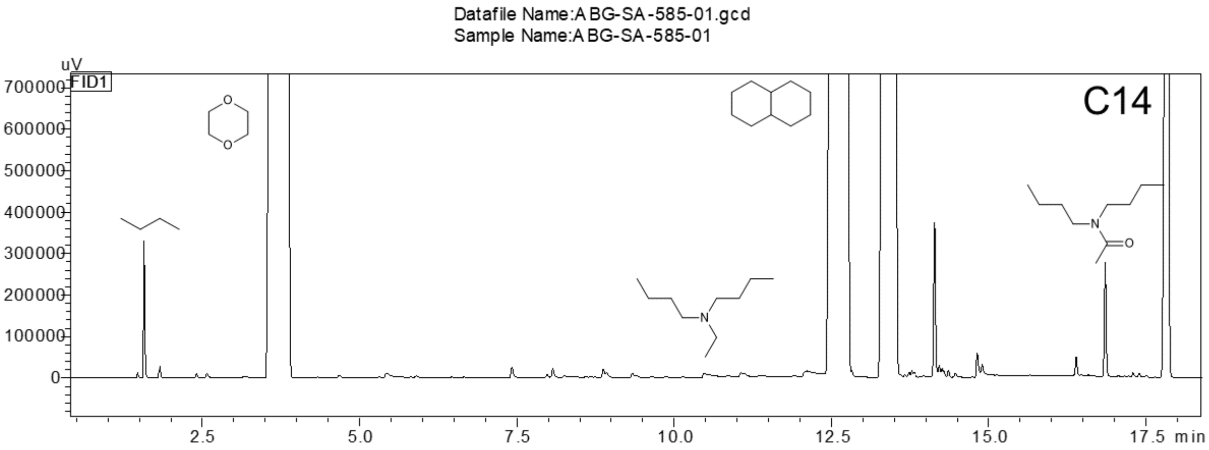

Figure S50. Chromatogram corresponding to the data of Table 2, Substrate **9**, 4 h.

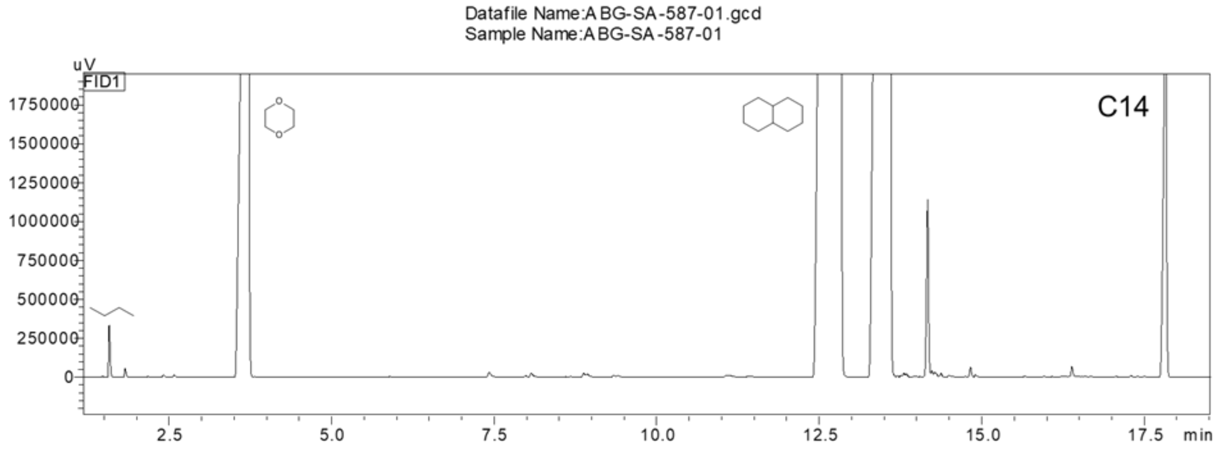

Figure S51. Chromatogram corresponding to the data of Table 2, Substrate **9**, 16 h.

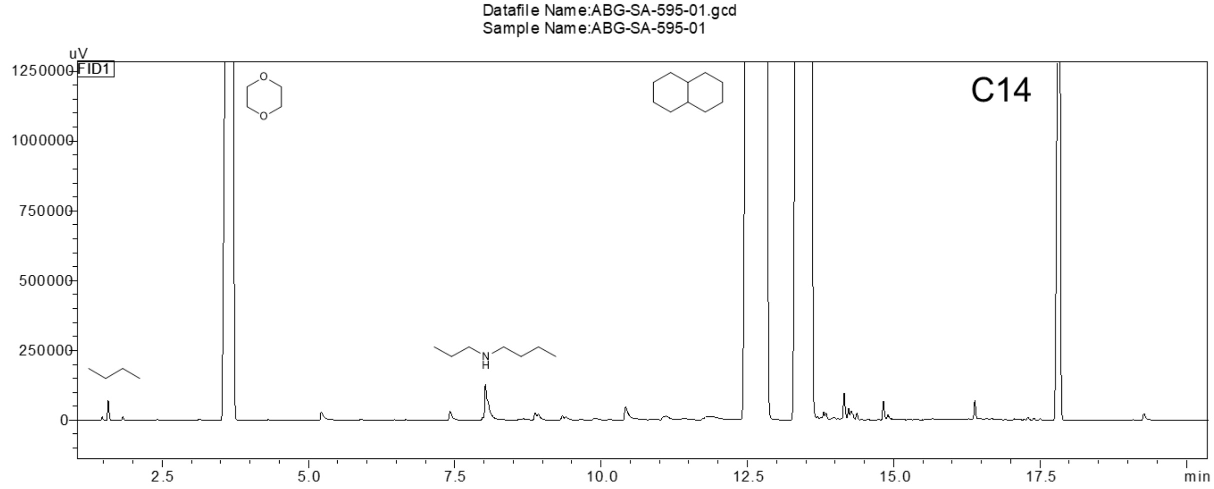

Figure S52. Chromatogram corresponding to the data of Table 2, Substrate **10**.

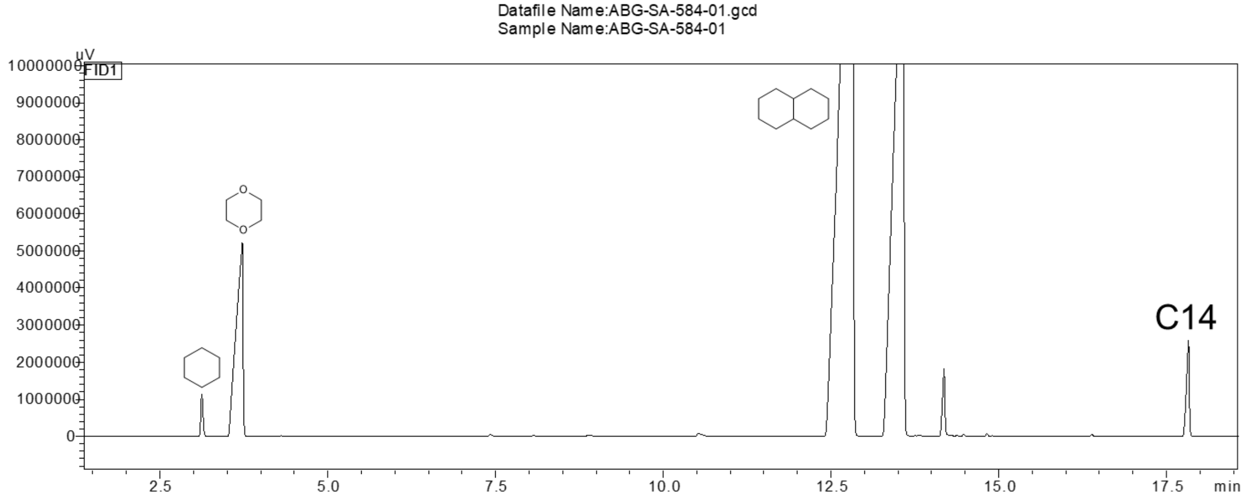

Figure S53. Chromatogram corresponding to the data of Table 2, Substrate **11**, 16 h.

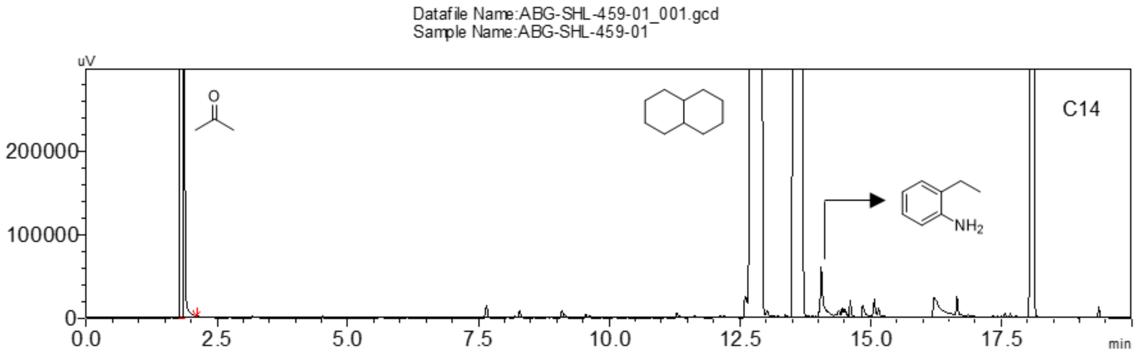

Figure S54. Chromatogram corresponding to the data of Table 2, Substrate **12** (72 mT, 4 h).

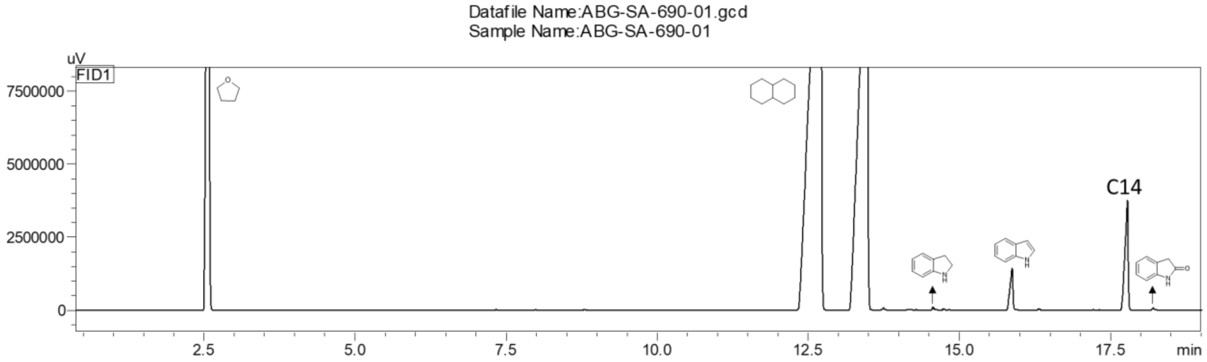

Figure S55. Chromatogram corresponding to the data of Table 2, Substrate **12** (60 mT, 16 h).

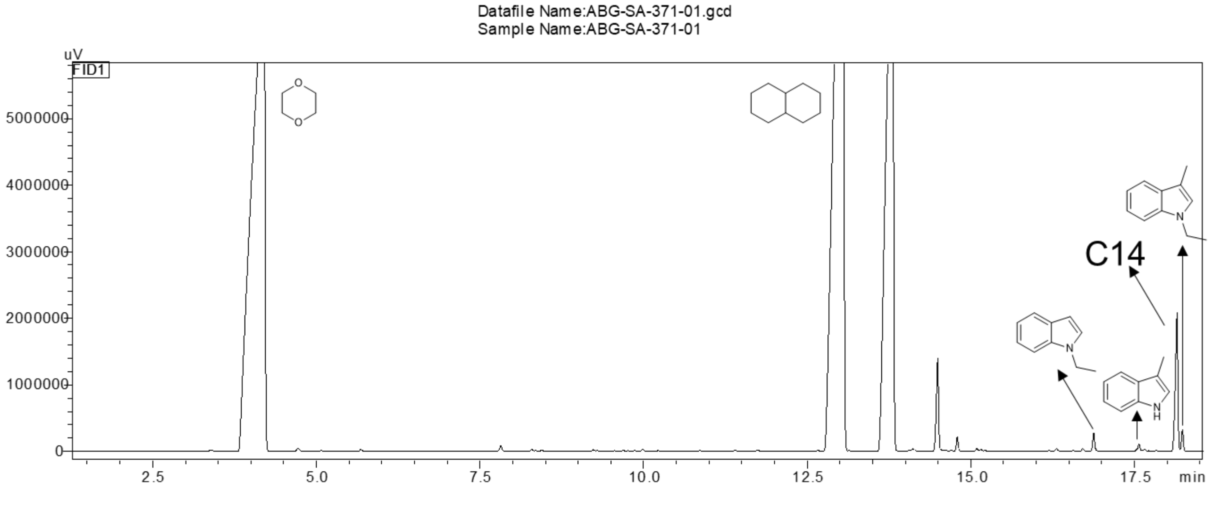

Figure S56. Chromatogram corresponding to the data of Table 2, Substrate **13**.

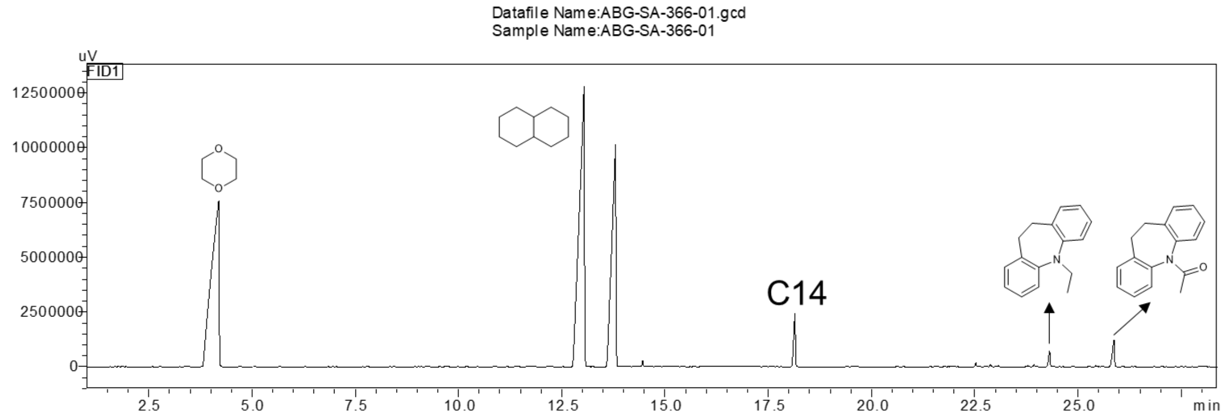

Figure S57. Chromatogram corresponding to Table 2, Substrate **14.**

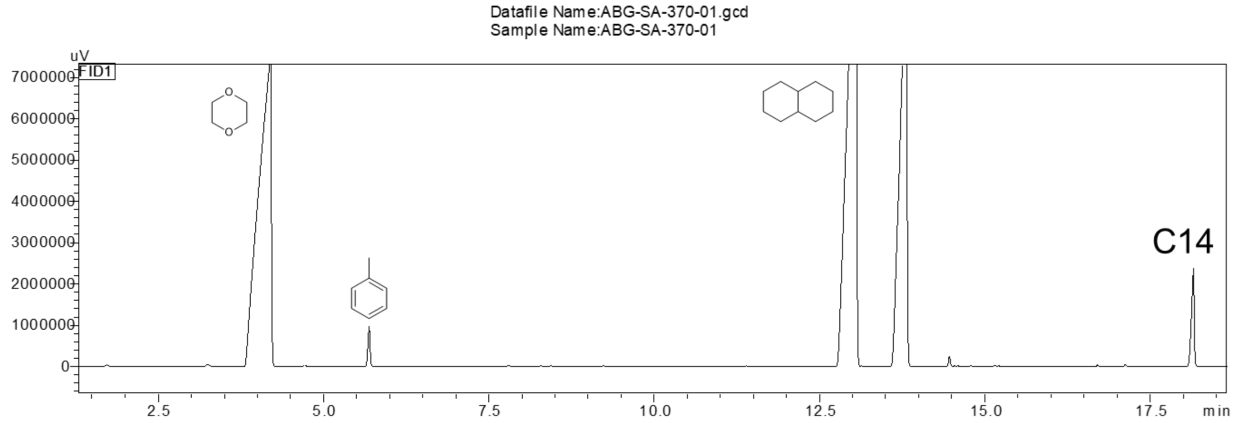

Figure S58. Chromatogram corresponding to the data of Table 2, Substrate **15**.

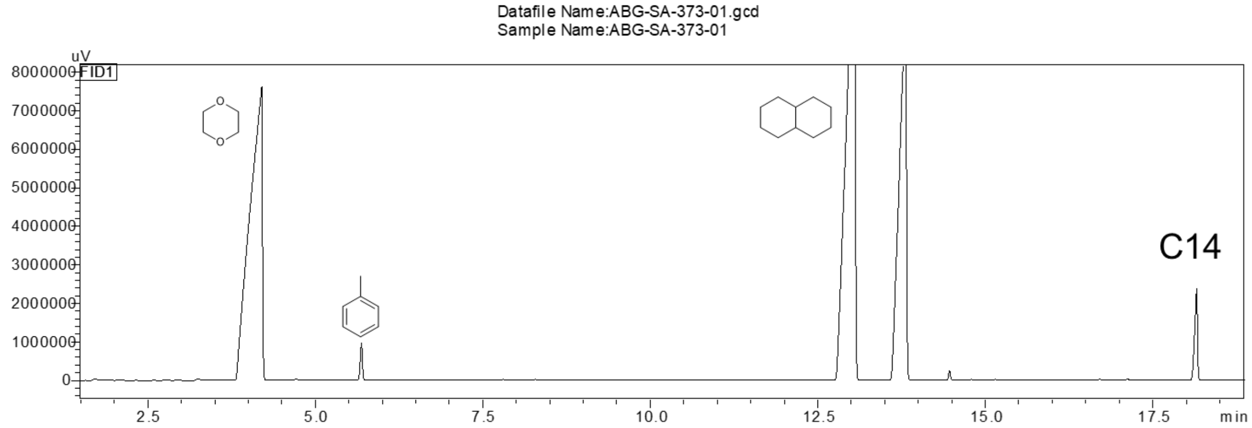

Figure S59. Chromatogram corresponding to the data of Table 2, Substrate **16**.

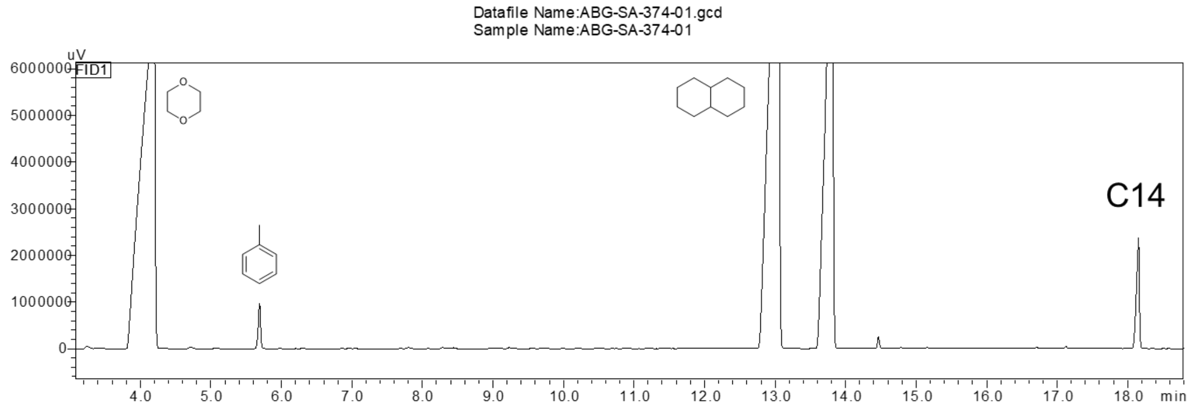

Figure S60. Chromatogram corresponding to the data of Table 2, Substrate **17**.

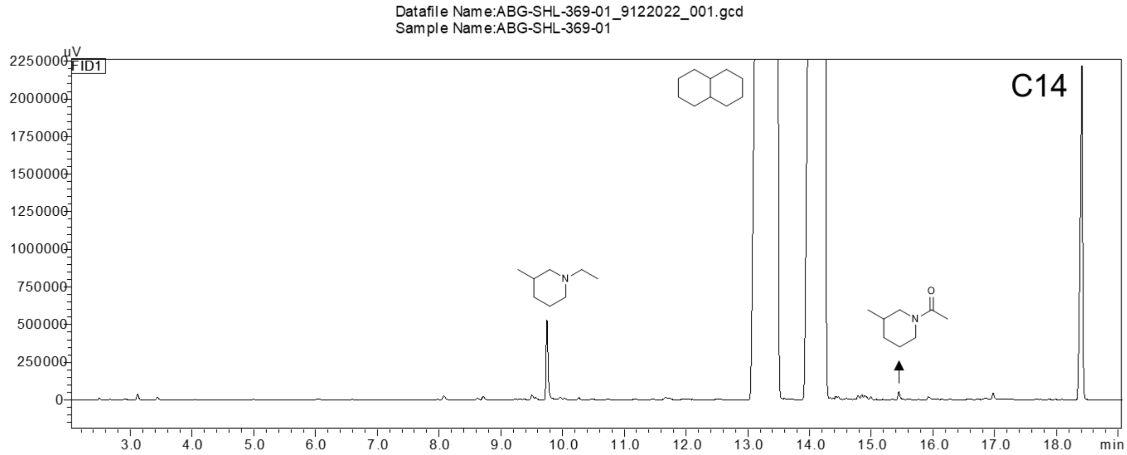

Figure S61. Chromatogram corresponding to Table S5, entry 1.

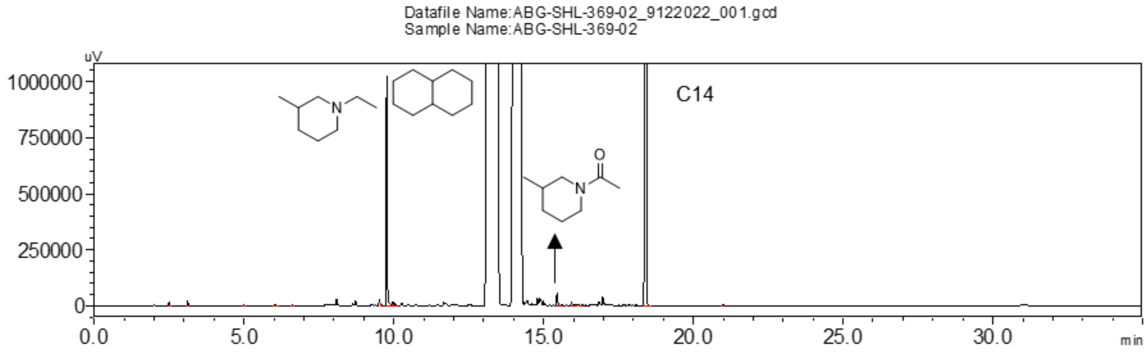

Figure S62. Chromatogram corresponding to Table S5, entry 2.

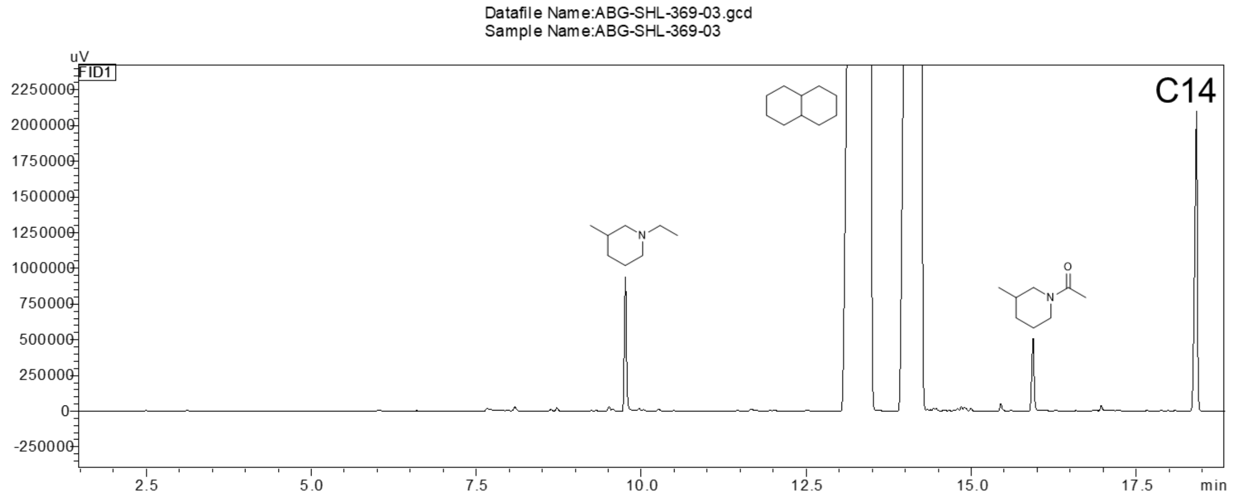

Figure S63. Chromatogram corresponding to Table S5, entry 3.

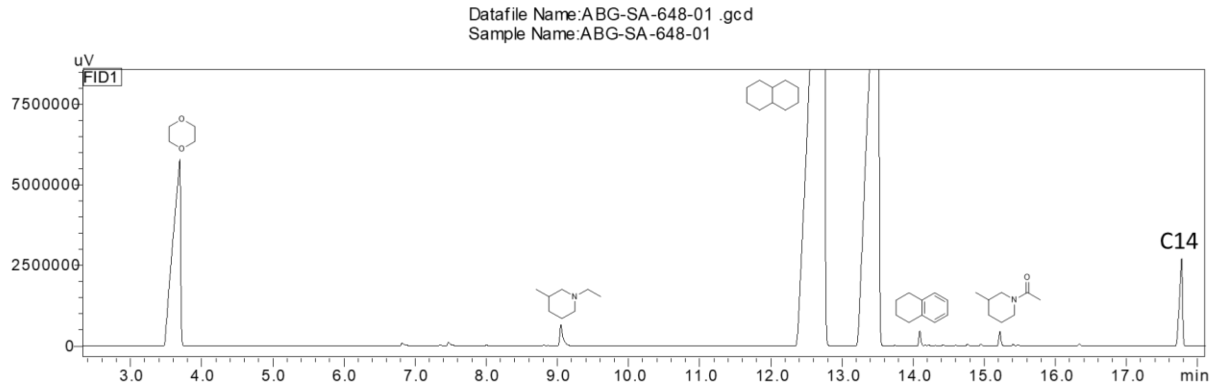

Figure S64. Chromatogram corresponding to the data of Figure S8a, cycle 1.

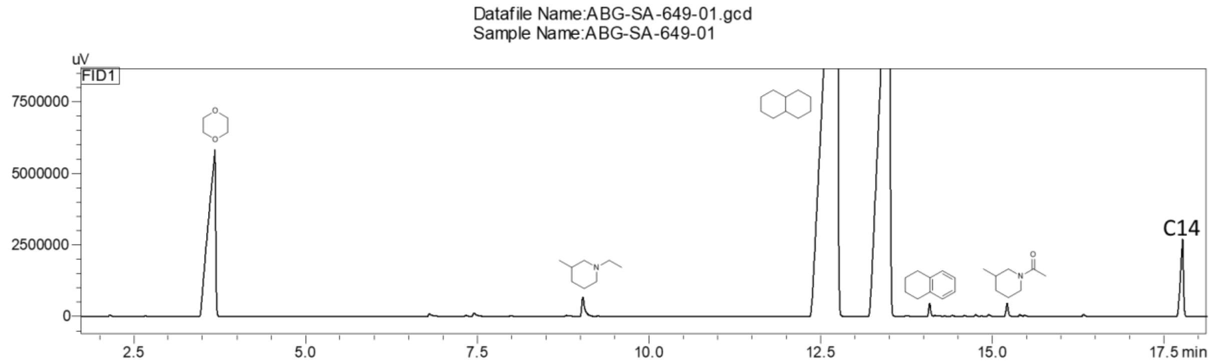

Figure S65. Chromatogram corresponding to the data of Figure S8a, cycle 2.

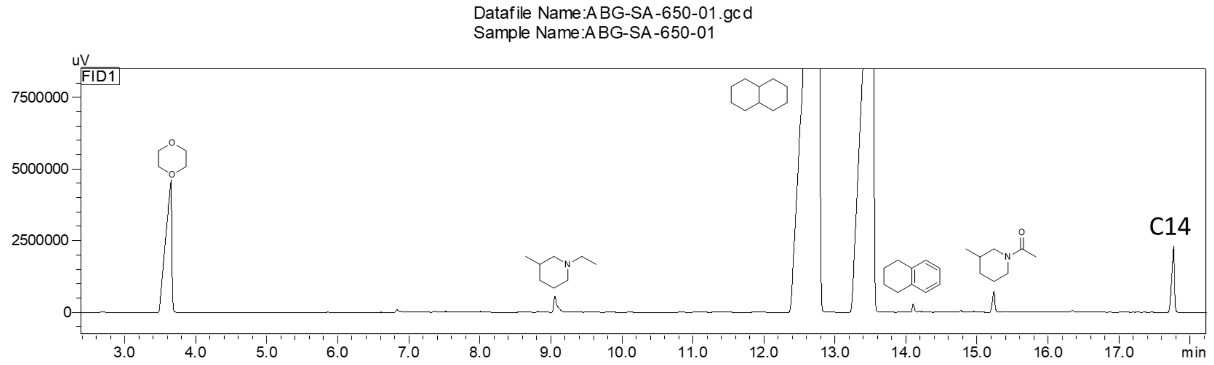

Figure S66. Chromatogram corresponding to the data of Figure S8a, cycle 3.

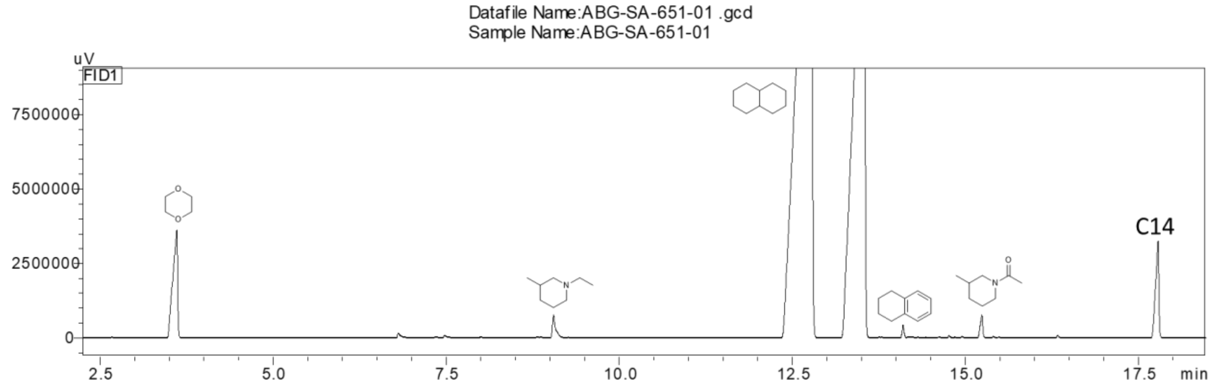

Figure S67. Chromatogram corresponding to the data of Figure S8a, cycle 4.

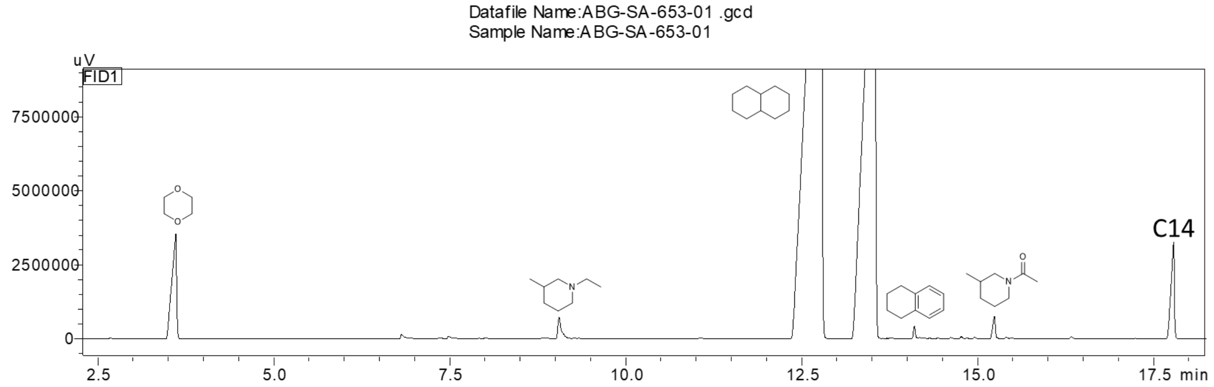

Figure S68. Chromatogram corresponding to the data of Figure S8a, cycle 5.

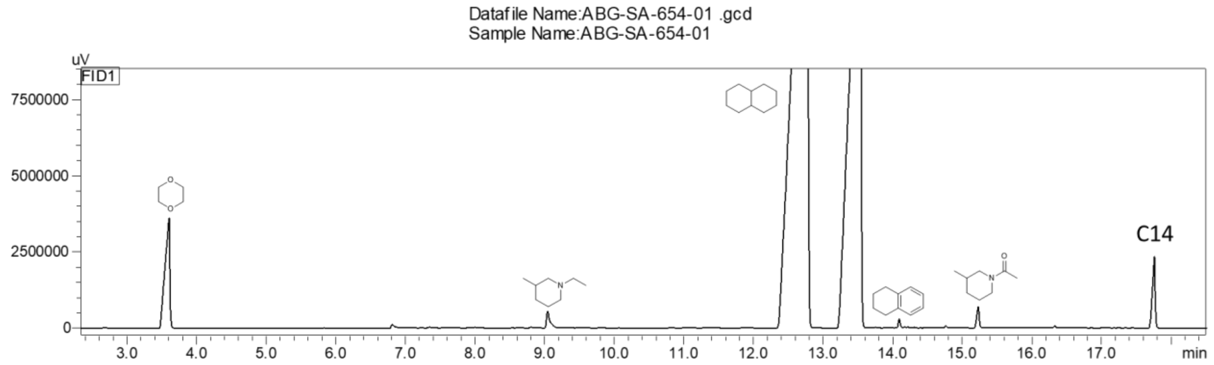

Figure S69. Chromatogram corresponding to the data of Figure S8a, cycle 6.

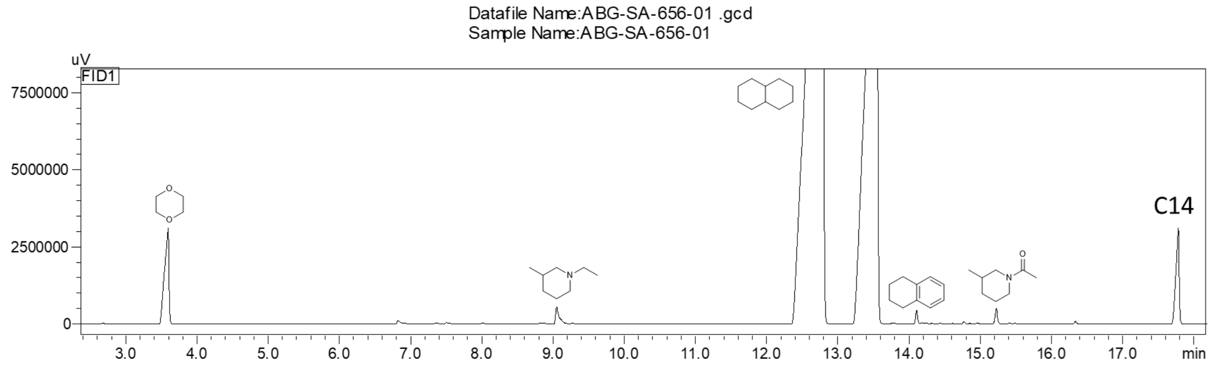

Figure S70. Chromatogram corresponding to the data of Figure S8a, cycle 7.

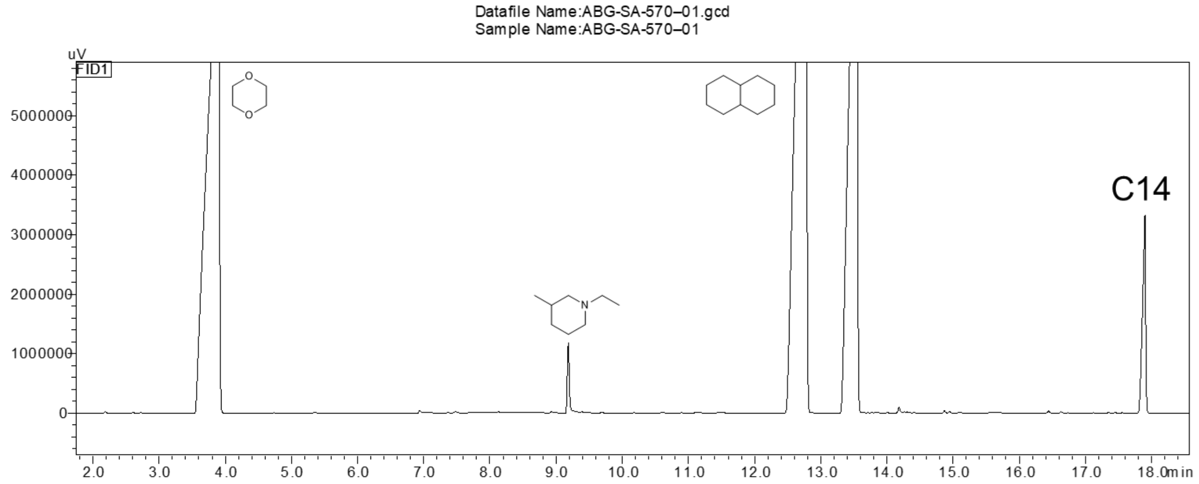

Figure S71. Chromatogram corresponding to the data of Figure S8b, cycle 1.

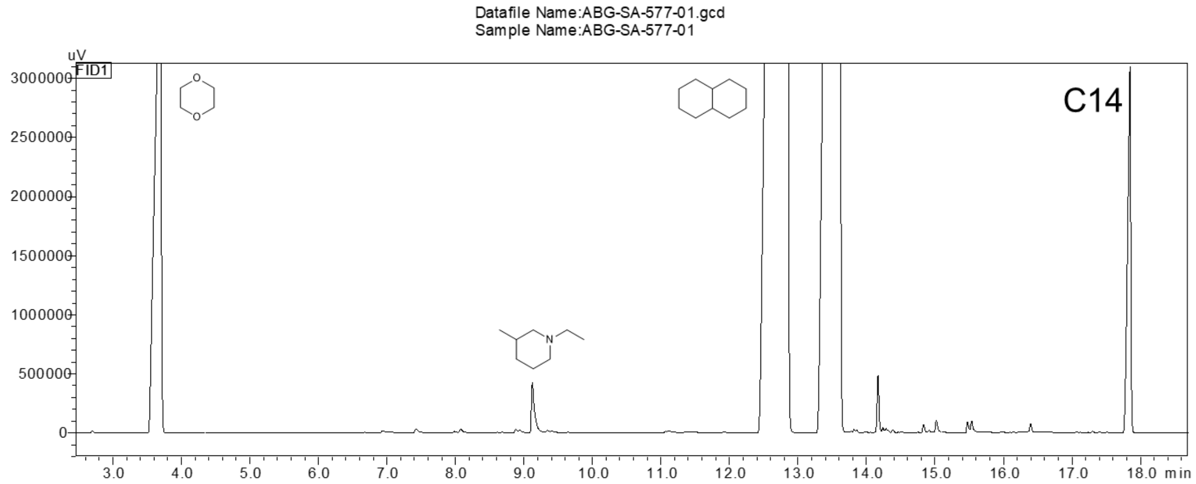

Figure S72. Chromatogram corresponding to the data of Figure S8b, cycle 2.

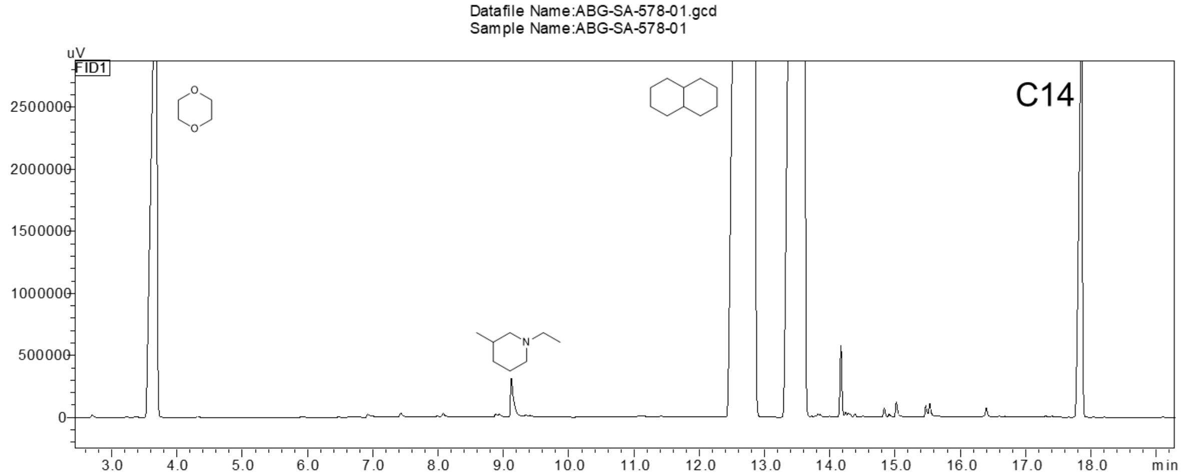

Figure S73. Chromatogram corresponding to the data of Figure S8b, cycle 3.

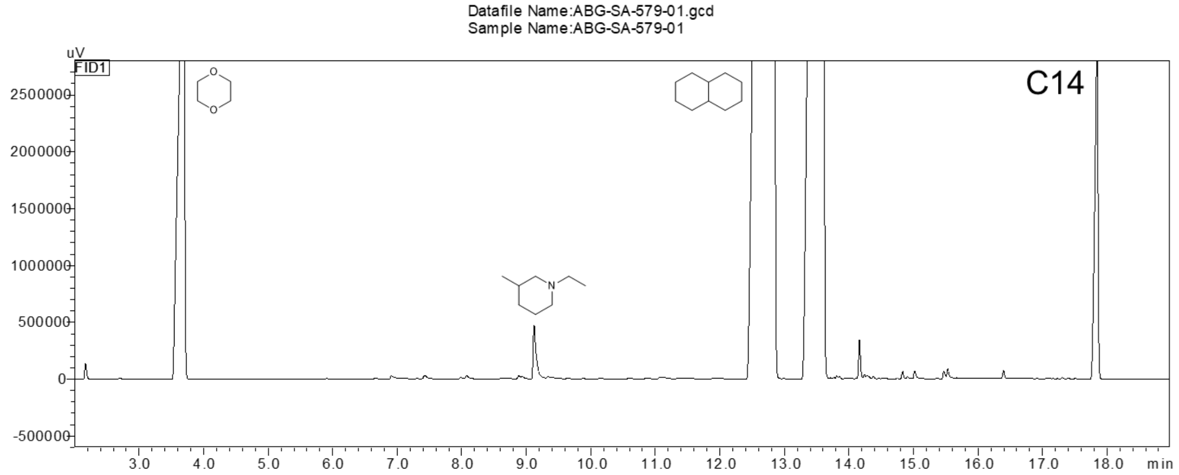

Figure S74. Chromatogram corresponding to the data of Figure S8b, cycle 4.

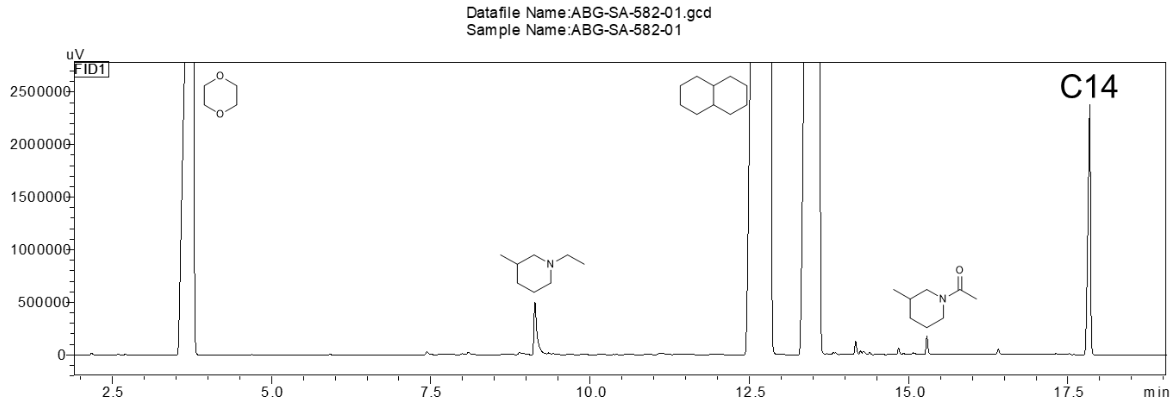

Figure S75. Chromatogram corresponding to the data of Figure S8b, cycle 5.

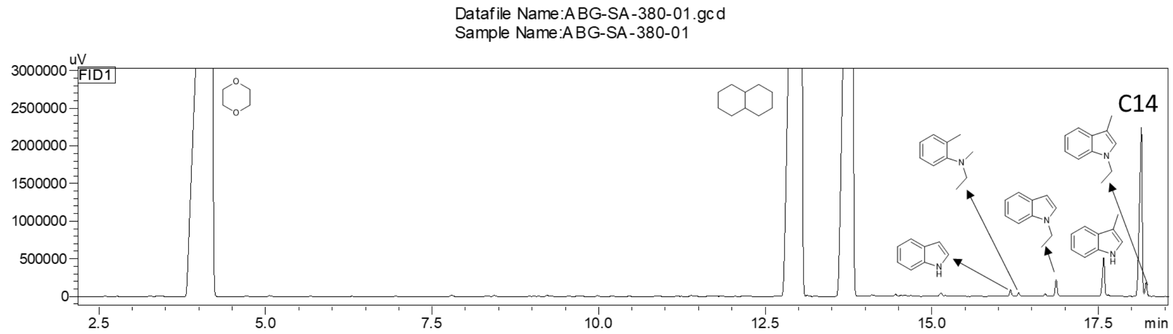

Figure S76. Chromatogram corresponding to the data of Figure S11.

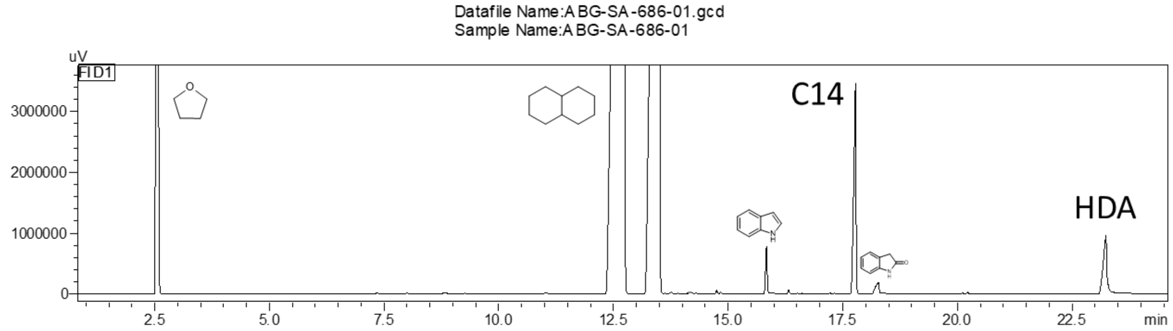

Figure S77. Chromatogram corresponding to the data of Figure S12 (4 h reaction).

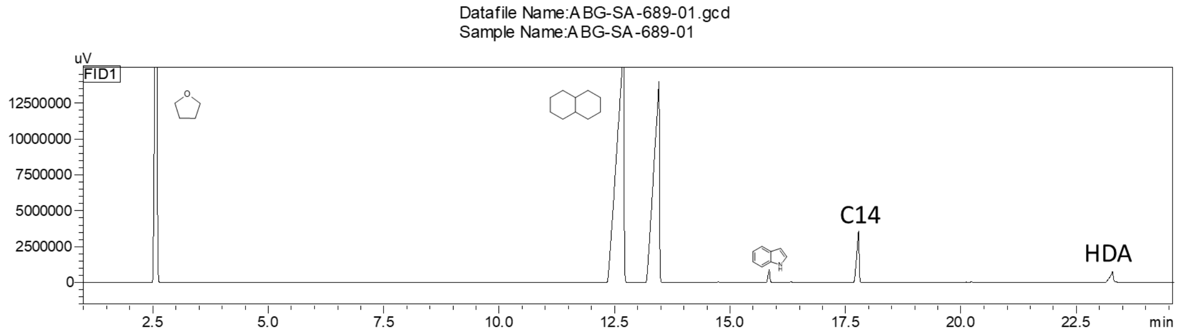

Figure S78. Chromatogram corresponding to the data of Figure S12 (8 h reaction).

References

1. D. L. J. Broere, I. Čorić, A. Brosnahan, P. L. Holland, *Inorg. Chem.* **2017**, *56*, 3140-3143.
2. E. P. Jahrman, W. M. Holden, A. S. Ditter, D. R. Mortensen, G. T. Seidler, T. T. Fister, S. A. Kozimor, L. F. J. Piper, J. Rana, N. C. Hyatt, M. C. Stennett, *Rev. Sci. Instrum.* **2019**, *90*, 024106.
3. E. Welter, R. Chernikov, M. Herrmann, R. Nemausat, *AIP Conf. Proc.* **2019**, *2054*, 040002.
4. B. Ravel, M. Newville, *J. Synchrotron Radiat.* **2005**, *12*, 537-541.
5. A. Bordet, L.-M. Lacroix, P.-F. Fazzini, J. Carrey, K. Soulantica, B. Chaudret, *Angew. Chem. Int. Ed.* **2016**, *55*, 15894.
6. K.-i. Shimizu, W. Onodera, A. S. Touchy, S. M. A. H. Siddiki, T. Toyao, K. Kon, *ChemistrySelect* **2016**, *1*, 736-740.
7. T. Mitsudome, K. Miyagawa, Z. Maeno, T. Mizugaki, K. Jitsukawa, J. Yamasaki, Y. Kitagawa, K. Kaneda, *Angew. Chem. Int. Ed.* **2017**, *56*, 9381-9385.
8. T. Toyao, S. M. A. H. Siddiki, Y. Morita, T. Kamachi, A. S. Touchy, W. Onodera, K. Kon, S. Furukawa, H. Ariga, K. Asakura, K. Yoshizawa, K.-i. Shimizu, *Chem. Eur. J.* **2017**, *23*, 14848-14859
9. C. Hirosawa, N. Wakasa, T. Fuchikami, *Tetrahedron Lett.* **1996**, *37*, 6749-6752.
10. G. Beamson, A. J. Papworth, C. Philipps, A. M. Smith, R. Whyman, *J. Catal.* **2011**, *278*, 228-238.

11. G. Beamson, A. J. Papworth, C. Philipps, A. M. Smith, R. Whyman, *Adv. Synth. Catal.* **2010**, *352*, 869-883
12. M. Stein, B. Breit, *Angew. Chem. Int. Ed.* **2013**, *52*, 2231-2234.
13. Y. Zhang, L. Li, F. Liu, H. Qi, L. Zhang, W. Guan, Y. Liu, A. Wang, T. Zhang, *ACS Catal.* **2022**, *12*, 6302-6312.
